# Generation of stable integration‐free pig induced pluripotent stem cells under chemically defined culture condition

**DOI:** 10.1111/cpr.13487

**Published:** 2023-05-15

**Authors:** Qianqian Zhu, Fengchong Wang, Dengfeng Gao, Jie Gao, Guilin Li, Deling Jiao, Gaoxiang Zhu, Kaixiang Xu, Jianxiong Guo, Tianzhi Chen, Suying Cao, Minglei Zhi, Jinying Zhang, Yingjie Wang, Xiaowei Zhang, Danru Zhang, Yixuan Yao, Jian Song, Hong‐Jiang Wei, Jianyong Han

**Affiliations:** ^1^ State Key Laboratory of Animal Biotech Breeding, College of Biological Sciences China Agricultural University Beijing China; ^2^ State Key Laboratory for Conservation and Utilization of Bio‐Resources in Yunnan Yunnan Agricultural University Kunming Yunnan China; ^3^ Animal Science and Technology College Beijing University of Agriculture Beijing China; ^4^ College of Biological Sciences China Agricultural University Beijing China

## Abstract

Genome integration‐free pig induced pluripotent stem cells (iPSCs) bring tremendous value in pre‐clinical testing of regenerative medicine, as well as conservation and exploitation of endangered or rare local pig idioplasmatic resources. However, due to a lack of appropriate culture medium, efficient induction and stable maintenance of pig iPSCs with practical value remains challenging. Here, we established an efficient induction system for exogenous gene‐independent iPSCs under chemically defined culture condition previously used for generation of stable pig pre‐gastrulation epiblast stem cells (pgEpiSCs). WNT suppression was found to play an essential role in establishment of exogenous gene‐independent iPSCs. Strikingly, stable integration‐free pig iPSCs could be established from pig somatic cells using episomal vectors in this culture condition. The iPSCs had pluripotency features and transcriptome characteristics approximating pgEpiSCs. More importantly, this induction system may be used to generate integration‐free iPSCs from elderly disabled rare local pig somatic cells and the iPSCs could be gene‐edited and used as donor cells for nuclear transfer. Our results provide novel insights into potential applications for genetic breeding of livestock species and pre‐clinical evaluation of regenerative medicine.

## Graphical abstract

An overview of model presented by this study. Pig induced pluripotent stem cells (iPSCs) reprogrammed using retrovirus or episomal vectors under the 3i/LAF culture medium could maintain long‐term self‐renewal and pluripotency without exogenous genes. Using episomal reprogramming system, rare pig integration‐free iPSCs could be generated, which could be gene‐edited and served as donor cells for cloning.

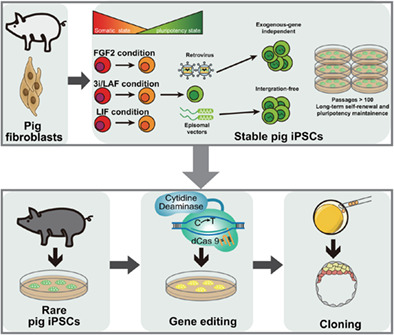

## 1 INTRODUCTION

Pluripotent stem cells (PSCs) have infinite capacity for self‐renewal and may develop into any tissue within the adult body. PSCs can be isolated from the embryonic inner cell mass, and are also known as embryonic stem cells (ESCs).^1^, ^2^ They may also be obtained by reprogramming somatic cells using exogenous factors, referred to as induced pluripotent stem cells (iPSCs).^3^ As iPSC technology is embryo‐independent and permits generation of individual‐specific stem cell types without the ethical issues of ESCs, it has broader applications in regenerative medicine and conservation of endangered germplasm resources.^4^, ^5^

The classical reprogramming process is mediated by retroviral vectors with defined factors—OCT3/4, SOX2, KLF4 and C‐MYC (collectively referred to as OSKM).^3^ Exogenous OSKM should be supplemented for amount of time and then silenced due to epigenetic changes in iPSCs with full pluripotency during the process of OSKM‐induced reprogramming.^6^ Indeed, retroviral silencing is an indicator of high‐quality iPSCs, which exhibit full pluripotent characteristics and maintain a pluripotent state in the absence of exogenous transgene expression.^7^ While exogenous transgenes delivered by retroviral vectors could effectively generate iPSCs,^8^ this method is not ideal given its low potential for clinical application. Vector integration‐free methods which avoid genomic contamination are thus being developed. The first report of integration‐free iPSCs was obtained via episomal vectors induction.^9^ Thereafter, reprogramming methods based on Sendai‐viral vectors,^10^ recombinant proteins^11^ and synthetic modified RNAs were developed.^12^ Among them, reprogramming by episomal vector is the most valuable procedure because of its dependability, convenience and clinical‐level safety.^13^ To date, episomal vector‐induced integration‐free iPSCs, similar to ESCs, have been obtained in humans,^9^ mice,^14^ and rats.^15^

Pigs and humans share numerous physiological and anatomical similarities, making pig iPSCs an excellent resource for pre‐clinical evaluation. Further, pig iPSCs can be employed not only for germplasm conservation of certain pig breeds, but also as valuable breeding materials. Over the past 13 years, efforts have been undertaken to establish pig iPSCs. However, developing exogenous gene‐independent and long‐term stable pig iPSCs is challenging in part due to the absence of a proper culture system. Recently, our team developed the 3i/LAF (GSK3β inhibitor CHIR99021, Tankyrase inhibitor IWR‐1, SRC inhibitor WH‐4‐023, LIF, Activin A and FGF2) system with selected cytokines and inhibitors based on transcriptomic features of pig E0‐E14 embryos at the single‐cell level. Stable pgEpiSCs (pre‐gastrulation epiblast stem cells) cultured in the 3i/LAF system could maintain stability over 240 passages, and yield cloned gene‐edited live piglets.^16^ That study has encouraged us to explore the reprogramming feasibility of the 3i/LAF system.

Here, we generated integration‐free iPSCs with long‐term self‐renewal capacity from pig somatic cells under our defined 3i/LAF culture system. We further proposed that inhibition of WNT signalling was required to establish exogenous gene‐independent iPSCs in pigs. This differs from mice and human iPSCs. The iPSCs cultured in the 3i/LAF system exhibited high similarity with pgEpiSCs. Furthermore, non‐integrated iPSCs reprogrammed from the somatic cells of elderly disabled or rare pigs may be genetically modified and used as donor cells for downstream nuclear transfer.

## 2 MATERIALS AND METHODS

A detailed description of all materials and methods can be found in Data S2 and Table S1: key resource table.

## 3 RESULTS

### 3.1 Establishment of integration‐free iPSCs under the 3i/LAF culture system

**FIGURE 1:**
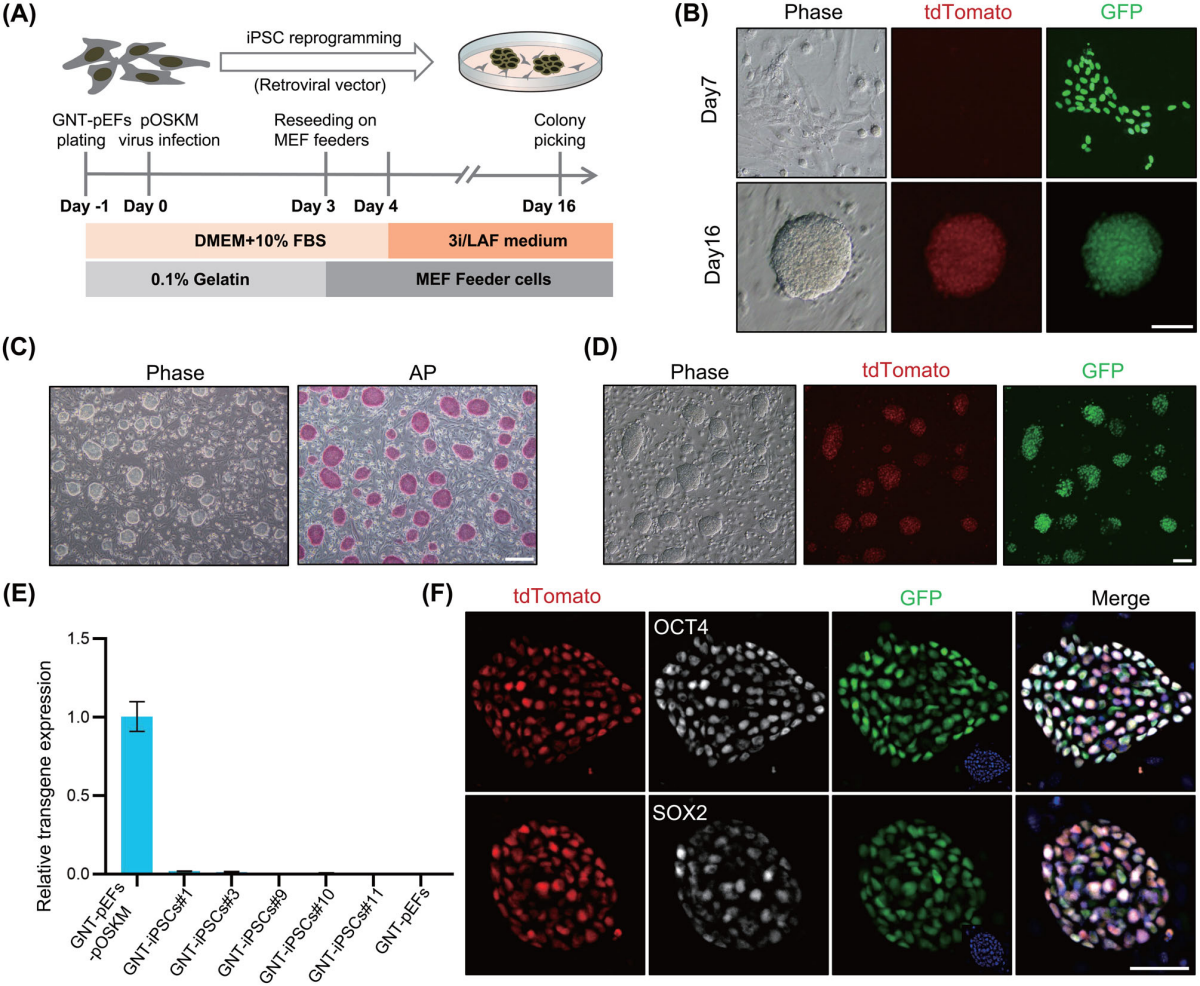
Generation of retrovirus‐silenced iPSCs from GNT‐pEFs using the 3i/LAF system. (A) Schematic illustration of the generation of pig iPSCs using a retroviral system. pOSKM, pig OCT3/4, SOX2, KLF4 and C‐MYC. (B) The process of generation of GNT‐iPSCs. Scale bar, 100 μm. (C) Colony morphology and AP staining of GNT‐iPSCs. Scale bar, 200 μm. (D) The GNT‐iPSCs showed uniform tdTomato fluorescence. Scale bar, 100 μm. (E) qPCR using primer specifically detecting viral transcript in GNT‐iPSCs. The GNT‐pEFs infected with the pOSKM virus for 3 days were used as control. The error bar indicates ± SD (*n* = 3, independent experiments). (F) IF staining of the pluripotency markers OCT4 and SOX2 in GNT‐iPSCs. Scale bar, 50 μm. The experiments in B, C, D and F were repeated independently three times with similar results. iPSCs, induced pluripotent stem cells; pEFs, pig ear fibroblast cells; pOSKM, pig OCT3/4, SOX2, KLF4 and C‐MYC; qPCR, quantitative RT‐PCR.

To investigate the iPSC induction capacity of the 3i/LAF defined culture medium, we employed a traditional retrovirus system with pig OSKM factors to carry out the iPSC induction program.^17^ Pig ear fibroblast cells derived from a GNT (GFP insertion, NANOG‐tdTomato knock in and *TYR* knock out)‐pgEpiSC‐cloned piglet (GNT‐pEFs) were used as initial cells,^16^ providing visualization of endogenous pluripotent gene NANOG expression (Figure S1A,B). The schematic illustration of the generation of pig iPSCs in 3i/LAF medium is shown in Figure 1A. A dramatic change in shape occurred in the fibroblast cells 7 days after infection (Figure 1B) and NANOG‐tdTomato‐positive colonies appeared at about 9 days post‐infection (Figure S1C). On 16th day, compact and plump cell colonies with domed shape and smooth edges, expressing active NANOG‐tdTomato fluorescence could form (Figure 1B). On average, 85 colonies were obtained from 4 × 10^4^ fibroblasts (reprogramming efficiency is 0.21%), with around 95.91% of these colonies showing expression of tdTomato fluorescence. The ratio of colonies with pluripotent reporter activation was almost comparble to those in mice.^18^ iPSCs reprogrammed from GNT‐pEFs (GNT‐iPSCs) were established with domed morphology, smooth edges and positive staining for AP (Figure 1C). In addition, these cells presented homogeneous NANOG‐tdTomato fluorescence (Figure 1D). Importantly, quantitative RT‐PCR (qPCR) analysis showed that the retroviral transgene was silenced in five randomly selected iPSC lines (GNT‐iPSCs#1, 3, 9, 10 and 11; Figure 1E). A comparison of total and endogenous pluripotency gene expression levels further confirmed silencing of the retroviral transgene (Figure S1D). Insertion of the retroviral vector in GNT‐iPSCs genome was validated by genomic PCR (Figure S1E). Immunofluorescent (IF) staining demonstrated the expression of other pluripotency markers, such as OCT4 and SOX2, as well as pluripotency surface markers including SSEA1, SSEA4, TRA‐1‐60 and TRA‐1‐81 (Figures 1F and S1F). These results suggest that the 3i/LAF system could be used to generate exogenous gene‐independent iPSCs, supporting the complete establishment of an endogenous pluripotency regulatory network.

**FIGURE 2:**
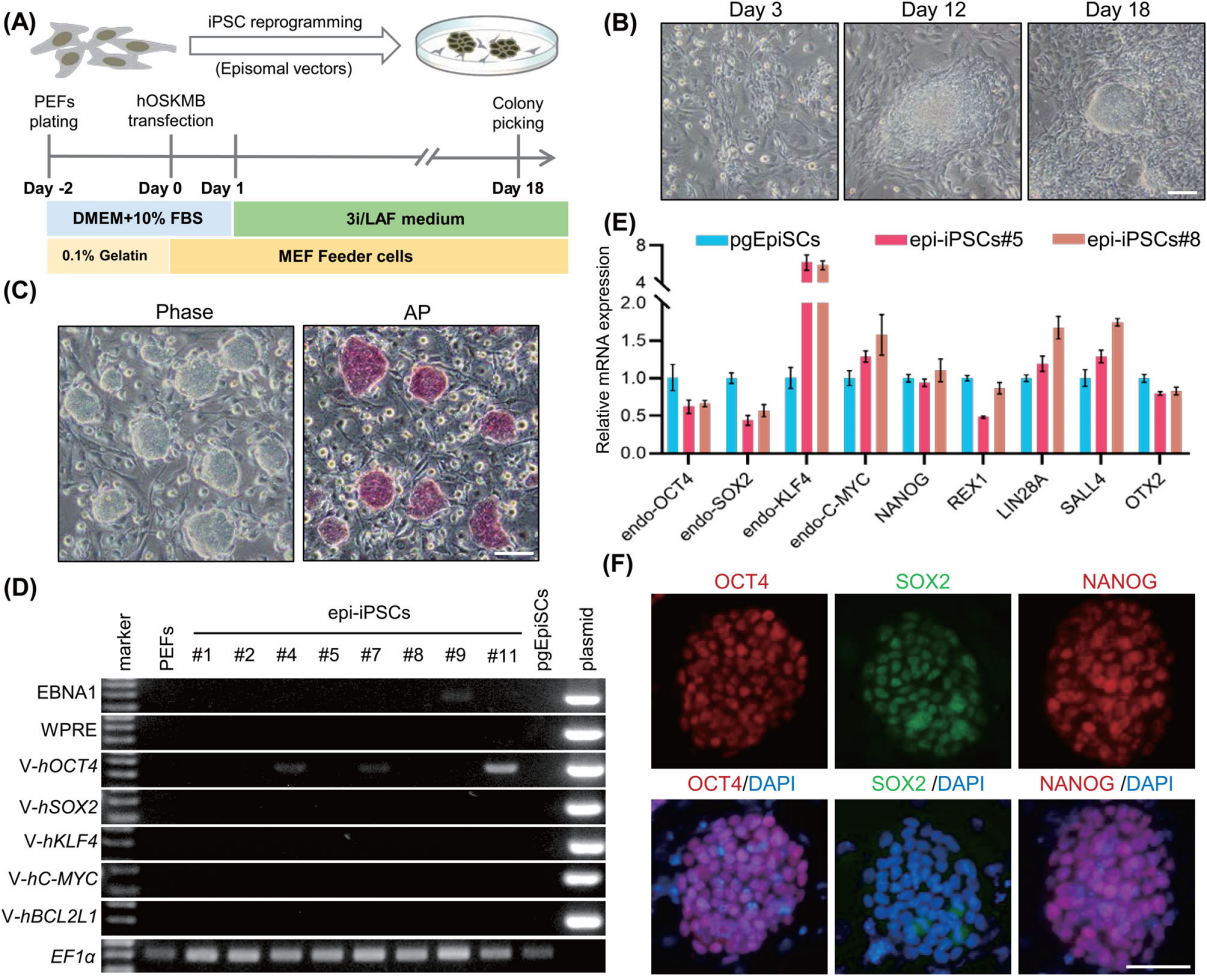
Establishment of integration‐free iPSCs under the 3i/LAF culture system. (A) Schematic illustration of the generation of pig iPSCs using an episomal system. (B) Reprogramming process of generating iPSCs. Scale bar, 100 μm. (C) Colony morphology and AP staining of epi‐iPSCs. Scale bar, 100 μm. (D) Genomic PCR using primers specific for episomal vectors in epi‐iPSCs at passage eight. V is an abbreviation of vector. (E) qPCR of pluripotency genes in epi‐iPSCs, the error bar indicates ± SD (*n* = 3, independent experiments). (F) IF staining of the key pluripotency markers OCT4, SOX2 and NANOG. Scale bar, 50 μm. The experiments in B, C, D and F were repeated independently three times with similar results. IF, immunofluorescent; iPSCs, induced pluripotent stem cells; PEFs, pig embryonic fibroblast cells; qPCR, quantitative RT‐PCR.

Noting the safety issues raised by genomes harbouring integrated exogenous sequences, the utility of pig iPSCs has been diminished in genetic breeding. Therefore, we attempted to build integration‐free pig iPSCs using previously reported episomal vectors under the 3i/LAF culture conditions.^19^ A schematic for the derivation of iPSCs using this episomal system (epi‐iPSCs) is shown in Figure 2A. Pig embryonic fibroblast cells (PEFs) developed an epithelial‐like appearance 3 days after electroporation. They gradually transformed into clumps and eventually formed ESC‐like colonies after ~18 days (Figure 2B). Although the reprogramming efficiency using episomal vectors was lower than that of traditional retroviral induction (the efficiency of reprogramming PEFs to iPSCs using retroviral vector (pMX‐iPSCs) was 0.42% ± 0.20%) (Figure S2A–E), 11 colonies were obtained from 1 × 10^5^ fibroblasts (reprogramming efficiency was 0.011%). Approximately 72.73% (8/11) of the colonies were successfully expanded into stable cell lines and the epi‐iPSC lines retained their dome‐shaped morphology and were positive for AP staining (Figure 2C). More importantly, 50% (4/8) of the iPSCs (epi‐iPSCs#1, 2, 5 and 8) at passage eight had no residual transcripts of the transgene integrated into the genome as validated by genomic PCR (Figure 2D), a frequency consistent with observations in humans.^13^ qPCR and IF staining demonstrated that epi‐iPSCs expressed pluripotency makers, as did the pMX‐iPSCs (Figures 2E,F and S2F,G). In conclusion, integration‐free iPSCs could be established using the 3i/LAF culture system.

### 3.2 Pluripotency features of 3i/LAF‐iPSCs

**FIGURE 3:**
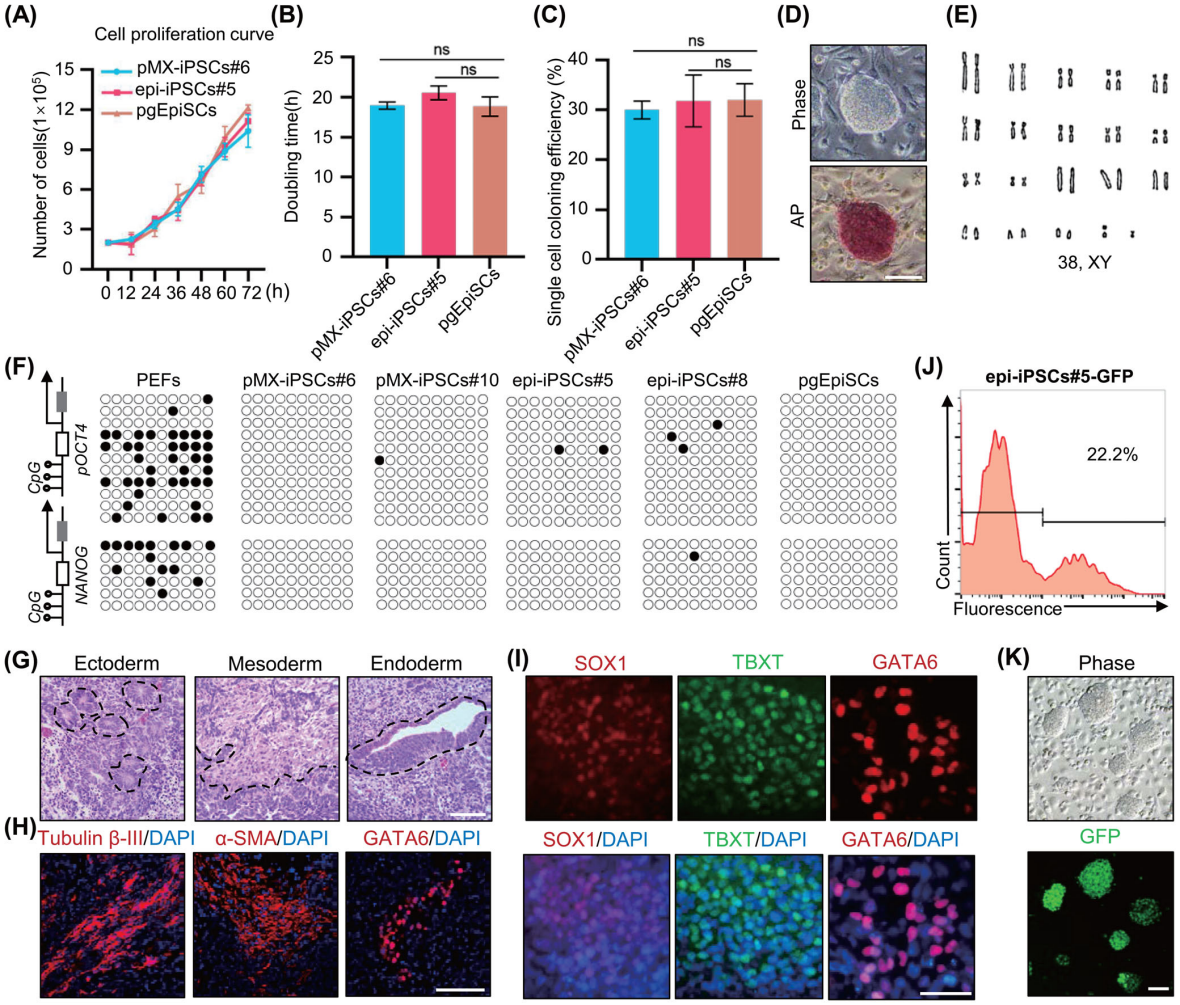
Pluripotency features of 3i/LAF‐iPSCs. (A) Cell proliferation curve of pMX‐iPSCs#6‐P32, epi‐iPSCs#5‐P31 and pgEpiSCs‐P35. P is an abbreviation of passage. (B) Population doubling time of pMX‐iPSCs#6‐P32, epi‐iPSCs#5‐P31 and pgEpiSCs‐P35. (C) Single cell coloning efficiency of pMX‐iPSCs#6‐P33, epi‐iPSCs#5‐P32 and pgEpiSCs‐P36. (D) Morphology and AP staining of epi‐iPSCs#5 at passage 93. Scale bar, 100 μm. (E) Karyotype analysis of epi‐iPSCs#5 at passage 95 (38, XY). (F) Bisulphite sequencing analysis of the CpG sites in the *OCT4* and *NANOG* promoter regions in PEFs, epi‐iPSCs#5/8, pMX‐iPSCs#6/10 and pgEpiSCs. (G) H&E staining of teratomas derived from epi‐iPSCs#8‐P20. Scale bar, 100 μm. (H) IF staining of the Tubulin β‐III, α‐SMA and GATA6 in teratomas derived from epi‐iPSCs#8‐P20. Scale bar, 100 μm. (I) IF staining of SOX1, TBXT and GATA6 in epi‐iPSCs#8‐P30 after directional induced differentiation. Scale bar, 100 μm. (J) Transfection efficiency of epi‐iPSCs#5‐P30 detected by ratio of GFP‐positive cells using flow cytometry. (K) GFP fluorescence of epi‐iPSCs#5‐GFP cell line, Scale bar, 100 μm. For A, B and C, the error bar indicates ± SD (*n* = 3, independent experiments). ns, *p* ≥ 0.05. The experiments in D, E, F, G, H, I, J and K were repeated independently three times with similar results. IF, immunofluorescent; iPSCs, induced pluripotent stem cells; PEFs, pig embryonic fibroblast cells; qPCR, quantitative RT‐PCR; pgEpiSCs, pig pre‐gastrulation epiblast stem cells.

We highlighted the features of 3i/LAF‐iPSCs, including pMX‐iPSCs and epi‐iPSCs. The doubling time for 3i/LAF‐iPSCs growth was ~19 h and the colony formation efficiency was about 30.92%, consistent with pgEpiSCs (Figure 3A–C).^16^ Importantly, the iPSCs could undergo long‐term passages while maintaining domed morphology, positive AP staining, expression of pluripotency genes and normal karyotypes (Figures 3D,E and S3A–D). Furthermore, the iPSCs with high passage numbers maintained genomic stability (Figure S3E–H). Bisulphite genomic sequencing analyses revealed hypomethylation of the *OCT4* and *NANOG* promoters of iPSCs (Figure 3F). Embryonic body (EB) in vitro differentiation assays demonstrated that 3i/LAF‐iPSCs could differentiate into three germ layers (Figure S3I). Teratoma formation assays confirmed the in vivo differentiation potential of 3i/LAF‐iPSCs as evidenced by haematoxylin and eosin (H&E) staining of the ectoderm (neural tissues), mesoderm (muscle tissues) and endoderm (gut epithelium tissues) and subsequent IF assays of ectoderm (Tubulin β‐III), mesoderm (α‐SMA) and endoderm (GATA6) (Figures 3G,H and S3J,K). Directional induced differentiation demonstrated that epi‐iPSCs could form the target germ layers when treated with special differentiation media (Figure 3I). Moreover, epi‐iPSCs had efficient transgenic ability through PiggyBac transposon‐mediated insertion of the NLS‐GFP cassette (Figure 3J,K). In summary, 3i/LAF‐iPSCs could undergo long‐term self‐renewal and exhibit the functional hallmarks of pluripotency.

### 3.3 3i/LAF‐iPSCs showed transcriptome similarity to pgEpiSCs

**FIGURE 4:**
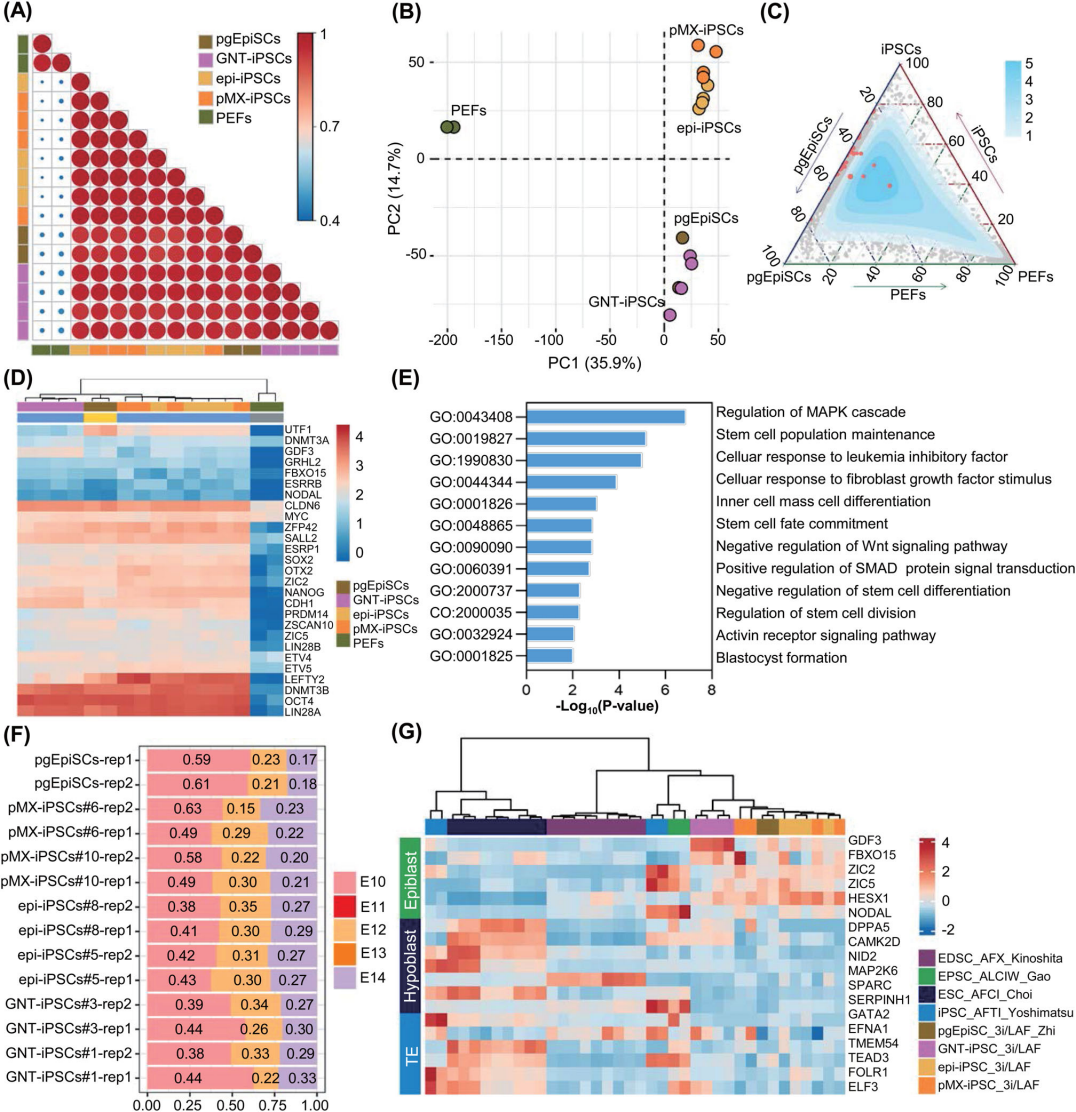
The similarity of 3i/LAF‐iPSCs and pgEpiSCs. (A) Pearson correlation coefficients of 3i/LAF‐iPSCs, pgEpiSCs and PEFs. (B) PCA analysis of 3i/LAF‐iPSCs, pgEpiSCs and PEFs. (C) Ternary plot of 3i/LAF‐iPSCs, pgEpiSCs and PEFs, with the red points representing pluripotency genes. (D) Heatmap of pluripotency genes in 3i/LAF‐iPSCs, pgEpiSCs and PEFs. (E) Gene ontology‐biological process terms of co‐upregulated differentially expressed genes in 3i/LAF‐iPSCs and pgEpiSCs, compared to PEFs. (F) Identity fractions of embryonic development stages for 3i/LAF‐iPSCs and pgEpiSCs. (G) Heatmap of classical marker genes of Epiblast, Hypoblast and TE in PSCs cultured under different media. iPSCs, induced pluripotent stem cells; PEFs, pig embryonic fibroblast cells; qPCR, quantitative polymerase chain reaction; pgEpiSCs, pig pre‐gastrulation epiblast stem cells; PCA, principal component analysis.

To gain a deep understanding of 3i/LAF‐iPSCs, we next performed RNA‐seq analyses of 3i/LAF‐iPSCs (GNT‐iPSCs, pMX‐iPSCs and epi‐iPSCs), pgEpiSCs and PEFs. When each of these was compared to PEFs, 3i/LAF‐iPSCs demonstrated remarkable consistency compared to pgEpiSCs, according to the calculated Pearson correlation coefficients (Figure 4A). Principal component analysis (PCA) further revealed the similarity of 3i/LAF‐iPSCs with pgEpiSCs in PC1, including majority of the pluripotency genes (Figures 4B and S4A). The ternary plot of total expression of genes demonstrated that the high‐density region was distributed between 3i/LAF‐iPSCs and pgEpiSCs, and most pluripotency genes were adjacent to the axis of pgEpiSCs and iPSCs (Figure 4C). Pluripotency gene expression levels were comparable between iPSCs and pgEpiSCs (Figure 4D). In comparison with PEFs, 1066 differentially expressed genes experienced upregulation in 3i/LAF‐iPSCs and pgEpiSCs, which were enriched in GO terms related to signalling pathway associated with pluripotency regulation, embryonic development and stem cell maintenance (Figures 4E and S4B). The PCA plot of in vivo E6‐E14 epiblast dataset and our PSC dataset together demonstrated that 3i/LAF‐iPSCs were clustered with pgEpiSCs, and they were comparable to E10 epiblasts (Figures 4F and S4C). Although the expression levels of a few genes related to stimulus response (*NR4A1*, *NR4A2*, *NR4A3*, *FOSB* and *CCN2*), metabolic processes (*FABP3*, *FN3KRP*, *DNPH1*, *CCN1*, *THBS1* and *RPL26*) and signal regulation (*ANXA1*, *RUSC1*, *SNX1*, *EGR4* and *DUSP2*) were different between iPSCs and pgEpiSCs, no significant pluripotency genes or stemness associated genes were detected (Figure S4D,E). In summary, 3i/LAF‐iPSCs are similar to pgEpiSCs at the transcriptional level.

The 3i/LAF‐PSCs (GNT‐iPSCs, pMX‐iPSCs, epi‐iPSCs and pgEpiSCs) were compared to previously published pig PSCs.^17^, ^20^, ^21^, ^22^, ^23^, ^24^, ^25^, ^26^, ^27^ PCA and Spearman correlation coefficients suggested that 3i/LAF‐PSCs displayed a degree of similarity to AFTI (Activin A, FGF2, TGFβ1 and Porcupine inhibitor IWP2)‐iPSCs,^21^ ALCIW (Activin A, LIF, CHIR99021, IWR‐1 and WH‐4‐023)‐PSCs,^20^ AFCI (Activin A, FGF2, CHIR99021 and IWR‐1)‐ESCs,^26^ and AFX (Activin A, FGF2 and Tankyrase inhibitor XAV939)‐EDSCs,^27^ although these cell lines all exhibited distinct alterations to transcription (Figure S4F,G). Further comparative analysis revealed that 3i/LAF‐PSCs expressed high levels of epiblast genes, including *GDF3*, *FBXO15*, *ZIC2*, *ZIC5* and *HESX1*, while expressing low levels of both hypoblast and trophectoderm (TE) genes (Figure 4G). To investigate the specific expression network that distinguished 3i/LAF‐PSCs from other PSC lines, weighted gene co‐expression network analysis (WGCNA) was applied. Genes contained in modules significantly associated with traits of 3i/LAF‐PSCs were enriched in translational initiation and regulation, cellular oxidant detoxification, gastrulation and reproductive system development (Figure S5A–C). By calculating the connectivity of these genes, 26 genes were identified as hub genes in 3i/LAF‐PSCs (Figure S5C). These hub genes displayed especially high expression in 3i/LAF‐PSCs, including *HESX1*, important for self‐renewal and pluripotency maintenance,^28^, ^29^ as well as *SOD2*, *ESD*, *OSTC*, *NSA2* and *PPM1K*, which reduce oxidative stress, regulate cell cycle and promote cell survival, respectively (Figure S5D).^30^, ^31^, ^32^, ^33^, ^34^ These results reflected the distinctive properties of 3i/LAF‐iPSCs in comparison to other PSCs. To summarize, 3i/LAF‐iPSCs exhibited striking similarities with pgEpiSCs.

### 3.4 The essential role of WNT inhibition in establishing exogenous gene‐independent iPSCs

**FIGURE 5:**
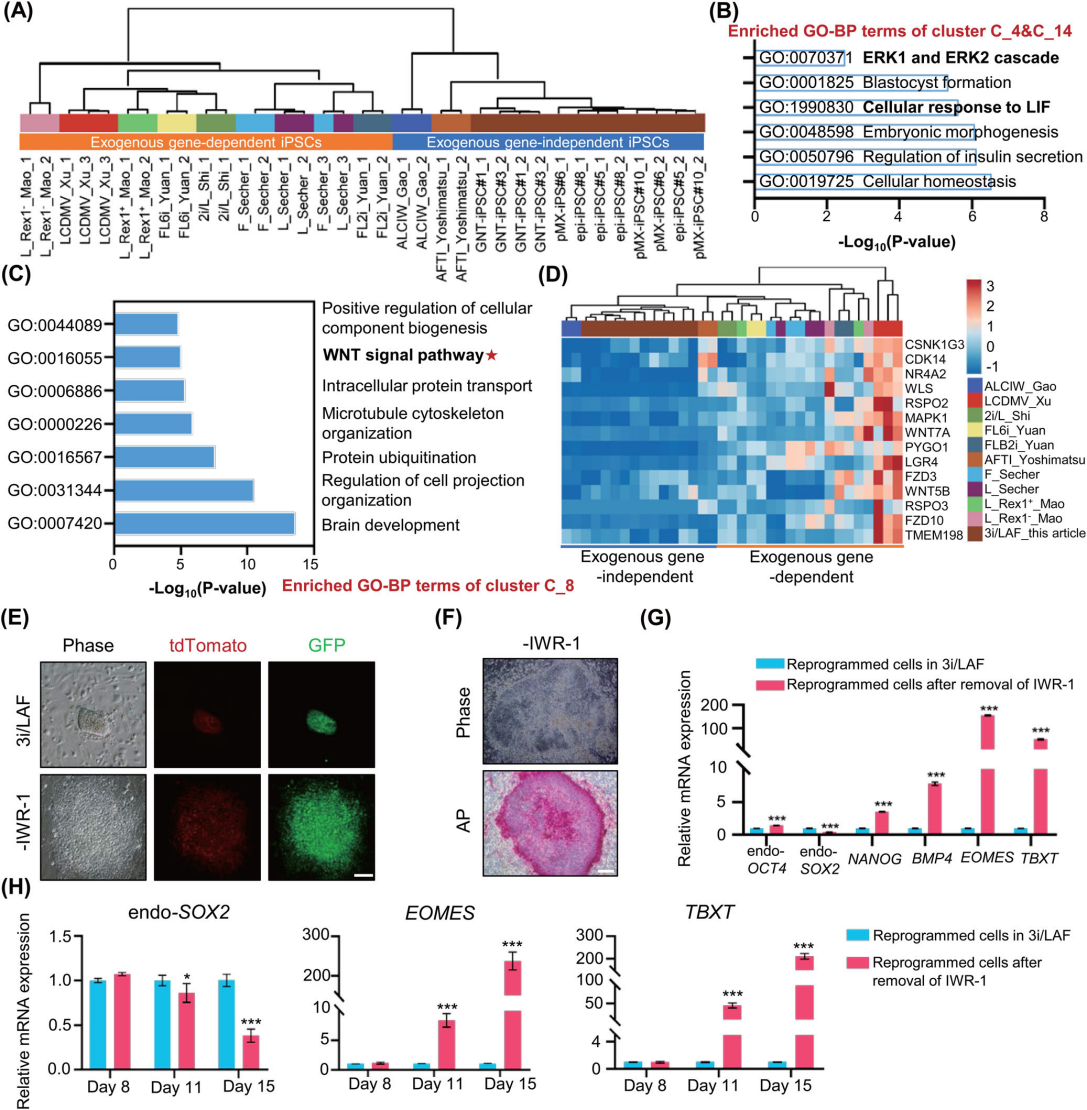
The essential role of WNT inhibition in establishing exogenous gene‐independent iPSCs. (A) Dendrogram obtained by applying ward.D2 algorithm to the Spearman correlation coefficient matrix of 3i/LAF‐iPSCs and public established pig iPSCs. (B,C) Gene ontology‐biological process enrichment of upregulated clusters (C_4 and C_14) (B) and downregulated cluster (C_8) (C) in exogenous gene‐independent iPSCs, compared with exogenous gene‐dependent iPSCs. (D) Heatmap of exogenous gene‐independent and exogenous gene‐dependent iPSCs illustrates the low expression of genes related to the WNT signalling pathway in exogenous gene‐independent iPSCs. (E) Morphology of NANOG‐tdTomato‐positive colonies cultured without IWR‐1 at day 18 of reprogramming. Scale bar, 100 μm. (F) Morphology and AP staining of reprogrammed cells cultured without IWR‐1 at day 20 of reprogramming. Scale bar, 200 μm. (G) qPCR of key pluripotency genes and representative mesendodermal differentiation related genes at 15 days post‐infection, the error bar indicates ± SD (*n* = 3, independent experiments), ***p < 0.001. (H) qPCR of endo‐*SOX2*, *TBXT* and *EOMES* at 8 days, 11 days, 15 days post‐infection. The error bar indicates ± SD (*n* = 3, independent experiments), *p < 0.05,***p < 0.001. The experiments in E and F were repeated independently three times with similar results.

We next sought to investigate the critical signalling pathway, which participates in establishment of exogenous gene‐independent iPSCs under the 3i/LAF culture conditions. Thereafter, we compared the bulk transcriptomes of exogenous gene‐independent iPSC lines, including 3i/LAF‐iPSCs generated here, ALCIW‐iPSCs,^20^ and AFTI‐iPSCs,^21^ with previously reported transcriptomes of exogenous gene‐dependent iPSC lines.^17^, ^22^, ^23^, ^24^, ^25^ Correlation coefficient matrix analysis illustrated that exogenous gene‐independent and exogenous gene‐dependent iPSC lines exhibited distinct transcriptional alterations (Figure 5A). Pluripotency genes, including *LEFTY2*, *ZSCAN10*, *UTF1*, *NANOG*, *ETV4*, *ETV5*, *ESRP1*, *ZFP42* and *PRDM14* were significantly upregulated in exogenous gene‐independent iPSCs compared to exogenous gene‐dependent iPSCs, suggesting that these genes may play a role in building pig pluripotency‐regulated networks (Figure S6A). 15 gene clusters exhibiting different gene expression tendencies were classified between exogenous gene‐independent and exogenous gene‐dependent iPSCs (Figure S6B). Gene ontology‐biological process (GO‐BP) enrichment analysis demonstrated that exogenous gene‐independent iPSCs upregulated the ERK1/ERK2 cascade signal and LIF response pathway features (Figure 5B). In contrast, these exogenous gene‐independent iPSCs downregulated the WNT pathway when compared with exogenous gene‐dependent iPSCs (Figure 5C,D). IWR‐1 in 3i/LAF system could attenuate Wnt/β‐catenin signalling. These results prompted us to explore the role of FGF2, LIF and IWR‐1 in pig iPSC reprogramming and pluripotency maintenance.

We first investigated the effects of FGF2 and LIF on iPSC reprogramming in the 3i/LAF culture system. Very few colonies appeared at day 20 post‐infection when the induction medium did not contain FGF2 (Figure S6C,D). In the absence of FGF2 on day 12 after induction, reprogrammed cells displayed limited proliferative capacity, comparing to 3i/LAF medium (Figure S6E). Although quite a few NANOG‐tdTomato‐positive colonies had been developed by day 18 of induction, the resultant cell clumps were looser and had difficulty establishing cell lines (Figure S6F). Previous report has demonstrated that pig iPSCs cultured in FGF2 medium could not silence exogenous genes.^35^ Therefore, FGF2 is a significant factor in encouraging the proliferation of reprogrammed cells during reprogramming but is not an essential contributor to silencing exogenous gene. We noticed that the induction efficiency of NANOG‐tdTomato‐positive colonies was significantly reduced after withdrawal of LIF; however, this removal had no effect on the retroviral transgene silencing of iPSCs (Figure S6C,G). These data suggest that FGF2 and LIF may not involve in mediation of exogenous gene silencing, but they are required for the efficient establishment of iPSCs.

IWR‐1 induces stabilization of the β‐catenin disruption complex via interaction with Axin, thus inhibiting the activity of the WNT/β‐catenin pathway.^36^ Although endogenous NANOG expression could be activated, reprogrammed cell colonies presented flattened morphology and weak AP staining when reprogramming was carried out in medium lacking IWR‐1 (Figure 5E,F). qPCR analysis confirmed expression of *NANOG* and endogenous *OCT4*. In contrast, endogenous *SOX2* was down‐regulated and mesendoderm marker genes, including *BMP4*, *EOMES* and *TBXT* were significantly up‐regulated, indicating the occurrence of mesendodermal differentiation when induced in a system lacking IWR‐1 (Figure 5G).^37^, ^38^ The NANOG‐tdTomato‐positive cells presented differentiated morphology, and it was difficult for them to form colonies after being picked for further culture (Figure S6H). These data indicate that withdrawal of IWR‐1 caused obvious differentiation and the inability to generate downstream iPSCs. Moreover, though reprogramming efficiency might reduce under media without Activin A, CHIR99021, or Vc, exogenous transgene‐independent iPSC lines could be obtained after removal of Activin A, CHIR99021, WH‐4‐023 or Vc and the iPSCs also presented NANOG‐tdTomato fluorescence and positive AP staining (Figure S6I–K). Previous study has reported that inhibition of WNT signalling is required to suppress mesendodermal differentiation during maturation phase of reprogramming in human.^37^ To find the critical stage for suppressing WNT signalling under the 3i/LAF system, qPCR was used to compare the expression of endo‐*SOX2*, *TBXT* and *EOMES* at different periods after withdrawal of IWR‐1. Results showed that at about Day 11, endo‐*SOX2* was downregulated and *TBXT*, *EOMES* exhibited significant upregulation when IWR‐1 was removed from 3i/LAF system. Therefore, it may be necessary to start suppressing the WNT signal on days 8–11 of reprogramming (Figure 5H). Combined with recently reported pig PSC culture media for further analysis,^16^, ^17^, ^26^, ^39^ it wasshown that systems containing a WNT inhibitor, such as AFI and AFCI, could generate retroviral transgene‐silenced iPSCs, whereas others could not (Figure S7A). The core pluripotency gene *OCT4* was highly expressed in retroviral transgene‐silenced iPSCs but not in others (Figure S7B). These results implied that expression of exogenous transgenes must be silenced to promote activation of endogenous pluripotency in pig iPSC, which is consistent with previous report in mice.^40^ Moreover, gene set enrichment analysis showed that WNT pathway was inhibited in PSCs under 3i/LAF and AFCI systems when WNT inhibitor IWR‐1 and WNT activator CHIR99021 were included in these culture media (Figure S7C,D). The phenomenon that CHIR99021 and IWR‐1 could work synergistically to mediate the inhibition of the WNT signalling pathway under 3i/LAF and AFCI system is consistent with previous reports in human, mouse and pig PSCs.^41^, ^42^ IF staining of β‐catenin in pMX‐iPSCs under 3i/LAF system demonstrated non‐nuclear localization state, which further verified the inhibition of canonical WNT signal (Figure S7E). qPCR illustrated that WNT signal related genes *PYGO1*, *LGR4* and *FZD10* showed lower expression levels in exogenous gene‐independent iPSC lines (AFI‐iPSCs, AFCI‐iPSCs and 3i/LAF‐iPSCs), comparing to exogenous gene‐dependent iPSC lines (Figure S7F). These data suggest the importance of WNT suppression in silencing retroviral transgene.

Interestingly, iPSCs cultured in AFI and AFCI media had a flattened shape rather than the domed shape of 3i/LAF‐iPSCs, and were corresponding to the morphology of iPSCs cultured after removal of WH‐4‐023 (Figure S7G,H). In addition, 3i/LAF‐iPSCs expressed lower levels of lineage markers than AFI‐iPSCs and AFCI‐iPSCs (Figure S7I). qPCR revealed that expression of epithelial‐mesenchymal transition (EMT)‐related genes such as *SRC*, *BMP2*, *BMP4*, *IGF2*, *WNT8A*, *EOMES*, *KRT8*, *SPARC* and *EPHA2* was increased in iPSCs cultured without WH‐4‐023 (Figure S7J). These results indicated that SRC inhibition could inhibit lineage specification through prevention of EMT, thereby allowing pig iPSCs to maintain stable domed morphology, as previously reported in mice.^43^ Moreover, the 3i/LAF culture system was more efficient at reprogramming and establishing iPSC lines (Figure S7K).

In conclusion, these results suggest that specific inhibition of WNT/β‐catenin is critical for generating exogenous gene‐independent iPSCs.

### 3.5 Targeted genetic manipulation using integration‐free rare local pig iPSCs

PSCs have improved performance compared to fibroblasts in germplasm preservation and genetic breeding due to their propensity toward long‐term proliferation. Furthermore, iPSCs have heightened advantages compared to ESCs in terms of preserving endangered or disabled pig breeds. Therefore, we investigated the feasibility of generating integration‐free iPSCs from an elderly, infertile, rare local Wujin fire hair line pig using the 3i/LAF system, as well as its potential for producing genetically modified animals.

**FIGURE 6:**
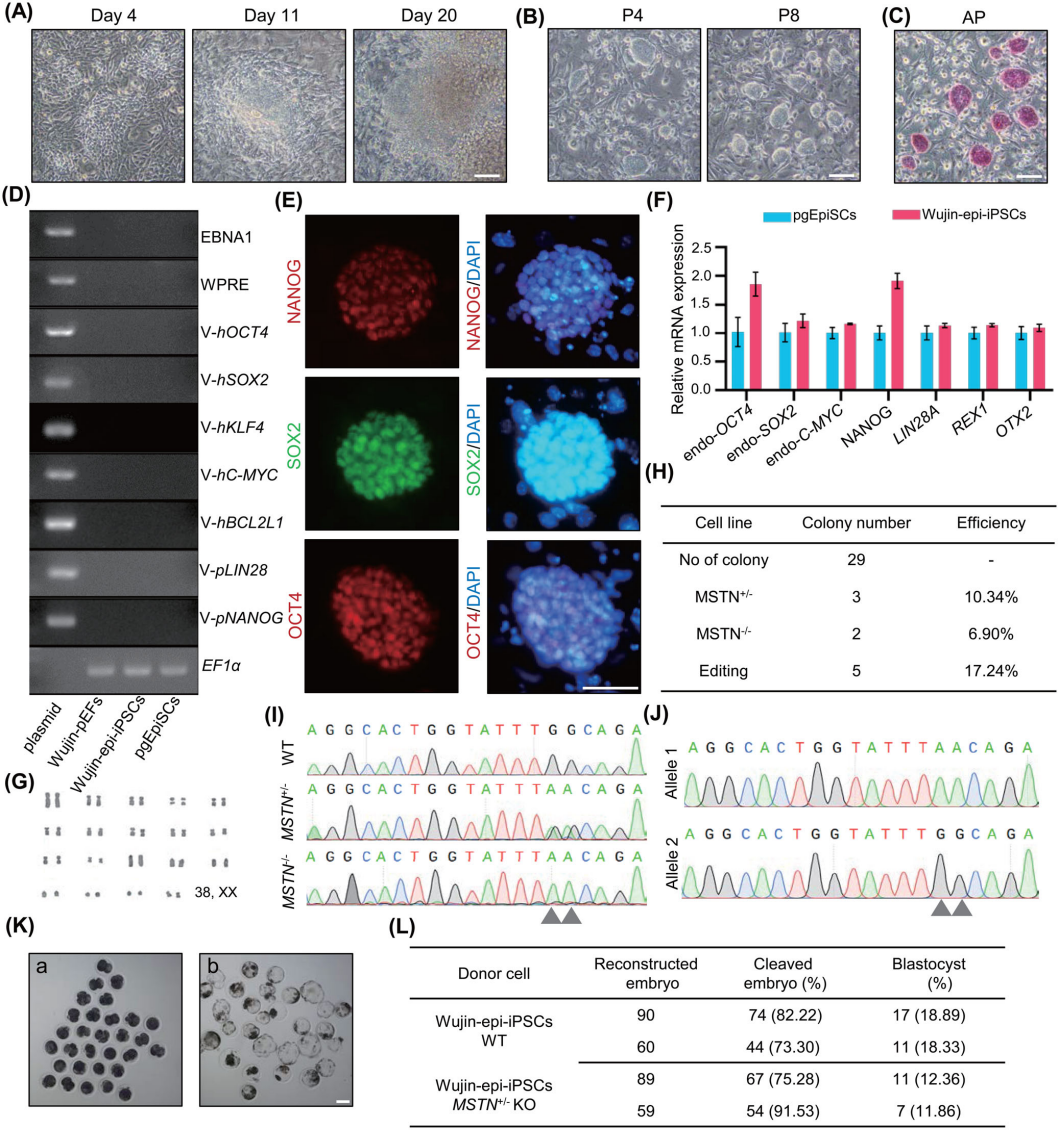
Targeted genetic manipulation using rare local pig integration‐free iPSCs. (A) Reprogramming process of generating Wujin‐epi‐iPSCs. Scale bar, 100 μm. (B) Colony morphology of Wujin‐epi‐iPSCs maintained from passage 4 through passage 8. Scale bar, 100 μm. (C) AP staining of Wujin‐epi‐iPSCs at passage 10. Scale bar, 100 μm. (D) Genomic PCR using primers related to episomal vector fragments in Wujin‐epi‐iPSCs at passage 8. (E) IF staining of OCT4, SOX2 and NANOG in Wujin‐epi‐iPSCs. Scale bar, 50 μm. (F) Pluripotency gene expression assay of Wujin‐epi‐iPSCs by qPCR, error bar indicates ± SD (*n* = 3, independent experiments). (G) Karyotype analysis of Wujin‐epi‐iPSCs at passage 15. (H) Statistics of heterozygous and homozygous mutation ratios for *MSTN* gene in Wujin‐epi‐iPSCs sub‐clones transfected with *MSTN* sgRNA. (I) Sanger sequence results for the target locus of *MSTN* in heterozygous and homozygous KO cell lines. (J) The allelic form of *MSTN* heterozygous KO cell lines. (K) Development of reconstructed embryos using Wujin‐epi‐iPSCs as donor cells for nuclear transfer. Development stage of cleaved embryos (a) and blastocysts (b). Scale bar, 100 μm. (L) Summary of cloning embryos development experiments (*n* = 2, independent experiments). The experiments in A, B, C, D, E and G were repeated independently three times with similar results.

The 10‐year‐old, infertile Wujin fire hair line pig ear fibroblast cells (Wujin‐pEFs) were used as initiating cells (Figure S8A,B). Putative iPSC colonies appeared after approximately 3 weeks of culturing under the 3i/LAF induction system (Figure 6A). Stable Wujin pig iPSCs (Wujin‐epi‐iPSCs) with domed morphology and positive AP staining were obtained (Figure 6B,C). Genomic PCR confirmed that there were no residual transcripts of the transgenes in the genome after passage eight (Figure 6D). qPCR and IF staining indicated the expression of pluripotency markers of Wujin‐epi‐iPSCs (Figure 6E,F). Moreover, the Wujin‐epi‐iPSCs were karyotypically normal (38, XX) (Figure 6G).

Myostatin (MSTN) regulates muscle tissue homeostasis through inhibition of skeletal muscle growth,^44^ and heterozygous *MSTN* deficiency can significantly increase the skeletal muscle growth rate in pigs while being less harmful to animal survivability.^44^, ^45^, ^46^ As local Wujin pigs have generally low lean meat percentages,^47^ *MSTN* was targeted to improve its production traits, making it more economically valuable. We then employed cytosine base editor to convert C to T, resulting in stop codon formation within exon 2 of the pig *MSTN* gene.^48^ Sanger sequencing was used to genotype 29 transfected sub‐colonies. Overall, effective C to T substitution was found in 17.24% (5/29) of the colonies, and 10.34% (3/29) of the colonies were heterozygous for the mutation (Figure 6H–J).

Wild type (WT) and heterozygous *MSTN* knock out (*MSTN*‐KO) Wujin pig iPSCs were then used as donor cells for generating cloned embryos. After injection and electrofusion of WT iPSCs with enucleated oocytes, 77.76% ± 4.46% of cleaved embryos were formed and 18.61% ± 0.28% of embryos developed into morphologically normal blastocysts (Figure 6K,L). Formation ratios of cleaved embryos and blastocyst from *MSTN* KO iPSCs were 83.41% ± 8.13% and 12.11% ± 0.25%, respectively (Figure 6L).

These results demonstrated that integration‐free pig iPSCs could be generated from elderly infertile, rare local pig somatic cells and that iPSCs could be successfully genetically manipulated and used as donor cells for nuclear transfer.

## 4 DISCUSSION

Pig iPSCs may be an excellent resource for pre‐clinical evaluation and genetic breeding. However, even after extensive and ongoing efforts since 2009, derivation of integration‐free and long‐term self‐renew pig iPSCs was still challenging due to lack of a proper culture system. Here, our findings create a novel integration‐free pig iPSC under our previous reported pgEpiSC culture condition^16^ and identify the role of WNT inhibition in pig iPSC generation, providing new possibilities for regenerative medicine and animal breeding.

Because epigenetic‐mediated silencing of foreign transgenes is a hallmark of full reprogramming, it may be used to test for presence of true iPSCs.^49^ Exogenous gene‐silenced iPSC lines can be created in mice using the LIF culture system^8^; however, human iPSCs with exogenous gene silencing were generated under the FGF2 culture condition.^50^ Using mice and humans reprogramming systems to reprogram pig iPSC resulted in a decades‐long failure to establish stable retroviral silencing pig iPSCs. It was proposed that there were interspecies differences in signalling pathways participated in pluripotency maintenance. Recently, WNT inhibition has been found to aid in self‐renewal of human naive ESCs,^51^ mouse formative PSCs^52^ and pig embryo‐derived stem cells.^20^, ^43^ We demonstrated that an additional WNT inhibitor supplementation may contribute to generation of retrovirally silenced pig iPSCs, allowing us to obtain integration‐free pig iPSCs. The role of WNT inhibition in establishment of pig iPSCs is different from human and mouse iPSCs. Significant mesendodermal differentiation occurred during reprogramming after WNT inhibitor removal in our 3i/LAF system. This might be due to the solely presence of WNT activator (CHIR99021), which promotes mesendodermal differentiation.^53^, ^54^ More importantly, addition of CHIR99021 in the presence of IWR1 could increase the accumulation of β‐catenin in the cytoplasm and promote the stability of stem cell.^42^ Regulation mechanisms of WNT inhibition in pig pluripotency maintenance and reprogramming process, as well as functional differences across species, require further investigation.

Other compounds with important functions were also included in the 3i/LAF culture condition, in addition to IWR‐1. FGF2 promotes reprogrammed cell proliferation, LIF exercises a considerable effect on establishment of pluripotency, and WH‐4‐023 mediates maintenance of domed morphology, all of which guarantee an efficient reprogramming system. CHIR99021 can cooperate with WNT inhibitors to improve cell line stability and expansion efficiency in mouse epiblast stem cells and human ESCs.^42^ CHIR99021 also promotes the proliferation of pig pgEpiSCs in the 3i/LAF culture medium.^16^ 3i/LAF may be an optimal culture system for establishment of pig iPSCs and may also be utilized to generate other livestock iPSCs.

3i/LAF‐iPSCs could preserve stability even after long‐term culturing, which may shorten the time required for gene editing using fibroblasts. Although cloned piglets may be obtained using in vitro differentiation‐mediated transgene silencing or in combination with epigenetic modification for iPSC nuclear transfer, the residual transgene fragments limited their value in production.^55^, ^56^ Here, we established an integration‐free iPSCs induction system, and genetically modified iPSCs could be used as donor cells for nuclear transfer, providing a useful strategy to conserve germplasm resources and improve breed characteristics. Furthermore, integration‐free pig iPSCs under chemically defined culture condition could be used as seed cells for cultured‐meat and pre‐clinical evaluation models.

Small‐molecule drugs were used to generate PSCs from mouse and human somatic cells,^57^, ^58^ which was thought to be safer in translational medicine. In this article, we show that the 3i/LAF culture system could effectively activate the endogenous pluripotent regulatory network. The construction of a pig iPSC chemical reprogramming system based on 3i/LAF medium will further increase the usefulness of pig iPSCs in genetic breeding, pre‐clinical assessment, and cultured‐meat production.

## AUTHOR CONTRIBUTIONS

*Conceived this study*: Jianyong Han. *Supervised the overall experiments*: Jianyong Han and Hong‐Jiang Wei. *Established all the iPSC lines*: Qianqian Zhu. *Performed cellular characteristics analysis*: Qianqian Zhu, Guilin Li, Gaoxiang Zhu, Yixuan Yao, Tianzhi Chen, Yingjie Wang, Danru Zhang and Jian Song. *Performed all the bioinformatics analysis*: Dengfeng Gao. *Generated cloning embryos*: Fengchong Wang, Deling Jiao, Kaixiang Xu and Jianxiong Guo. *Performed gene editing experiments*: Jie Gao and Xiaowei Zhang. *Helped with experiment design and discussions*: Suying Cao, Minglei Zhi and Jinying Zhang. *Wrote and reviewed the manuscript*: Jianyong Han, Qianqian Zhu, Dengfeng Gao and Guilin Li.

## FUNDING INFORMATION

This work was supported by the National Key R&D Program of China (2022YFD1302201 and 2016YFA0100202), Future Functional Food R&D Program of China Agricultural University (SJ2021002004), the National Natural Science Foundation of China (31970825 and 31772601), the Major Science and Technology Project of Yunnan Province (202102AA100009), the Chinese Universities Scientific Fund (2022TC018), the Plan 111 (B12008) and the Beijing Student's Innovation Training Program (S202210019012).

## CONFLICT OF INTEREST STATEMENT

The authors declare that they have no conflict of interest.

## Supporting information


**Figure S1.** Establishment of retrovirus‐silenced iPSCs by reprogramming GNT‐pEFs. Related to Figure 1.
**Figure S2.** Generation of retrovirus‐silenced iPSCs from PEFs.
**Figure S3.** Pluripotency characteristics of 3i/LAF‐iPSCs. Related to Figure 3.
**Figure S4.** RNA‐seq analysis of 3i/LAF‐iPSCs. Related to Figure 4.
**Figure S5.** The special transcriptome traits of 3i/LAF‐PSCs.
**Figure S6.** WNT inhibition promotes the establishment of exogenous gene‐independent iPSCs. Related to Figure 5.
**Figure S7.** 3i/LAF system is more stable in terms of reprogramming to establish pluripotency.
**Figure S8.** The state of ear fibroblasts derived from 10‐year‐old female rare local Wujin fire fair line pig. Related to Figure 6.

**Data S1.** Supporting information

**Table S1.** Key Resources Table

## Data Availability

All the RNA‐seq datasets generated in this study are summarized in Supporting information and had been deposited in the NCBI under accession GSE221892 (reviewer token: gfupmsqebjojbaf). Custom scripts used in the study can be downloaded from https://github.com/dfgao/pig.iPS.RNAseq-analysis. All other relevant data are available from the corresponding author on reasonable request.
