# Supplementary material for: Generation of stable integration‐free pig induced pluripotent stem cells under chemically defined culture condition
Source: Cell Prolif. 2023 May 15;56(11):e13487. doi: 10.1111/cpr.13487 (PMC10623960; doi:10.1111/cpr.13487)
Supplement: Supplementary file 1 — Figure S1. Establishment of retrovirus‐silenced iPSCs by reprogramming GNT‐pEFs. Related to Figure 1. Figure S2. Generation of retrovirus‐silenced iPSCs from PEFs. Figure S3. Pluripotency characteristics of 3i/LAF‐iPSCs. Related to Figure 3. Figure S4. RNA‐seq analysis of 3i/LAF‐iPSCs. Related to Figure 4. Figure S5. The special transcriptome traits of 3i/LAF‐PSCs. Figure S6. WNT inhibition promotes the establishment of exogenous gene‐independent iPSCs. Related to Figure 5. Figure S7. 3i/LAF system is more stable in terms of reprogramming to establish pluripotency. Figure S8. The state of ear fibroblasts derived from 10‐year‐old female rare local Wujin fire fair line pig. Related to Figure 6. [file CPR-56-e13487-s002.docx]

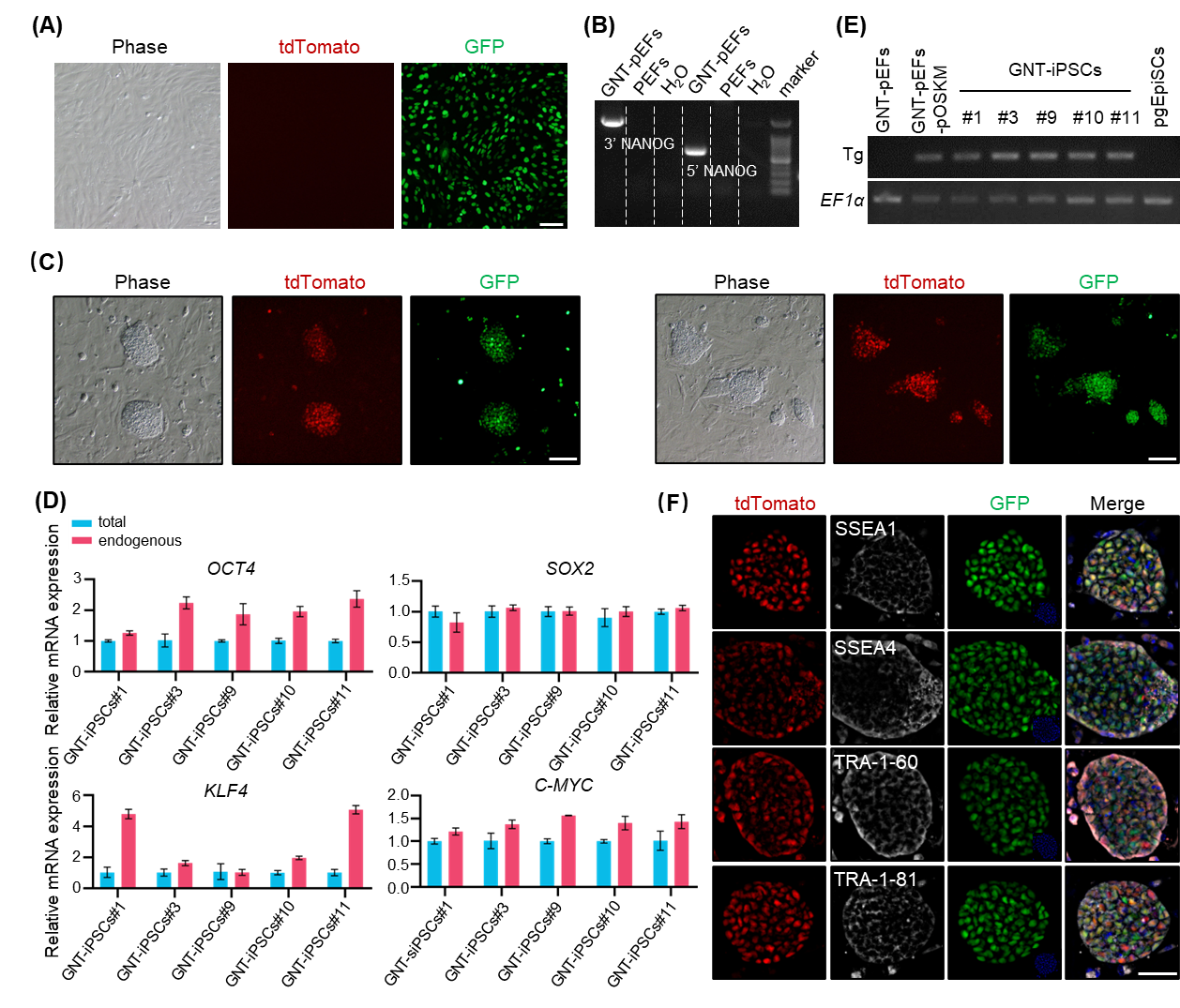


**Figure S1. Establishment of retrovirus-silenced iPSCs by reprogramming GNT-pEFs. Related to Figure 1.**

**A,** Morphology of derived GNT-pEFs. Scale bar, 100 μm.

**B,** Gel photos of PCR tests for NANOG-tdTomato knock-in. Wild type PEFs were used as control.

**C,** Morphology and fluorescence of reprogrammed cells 9 days post-infection. Scale bar, 100 μm.

**D,** qPCR using primers specific to both endogenous and transgenic transcripts (total) or endogenous (endo) mRNA in GNT-iPSCs. The error bar indicates ± SD (*n*=3, independent experiments).

**E,** Genomic PCR for retroviral vector in GNT-iPSCs. Tg is an abbreviation for transgene.

**F,** IF staining of the pluripotency surface markers SSEA1, SSEA4, TRA-1-60, and TRA-1-81 for GNT-iPSCs. Scale bar, 50 μm.

The experiments in A, B, C, E and F were repeated independently three times with similar results.


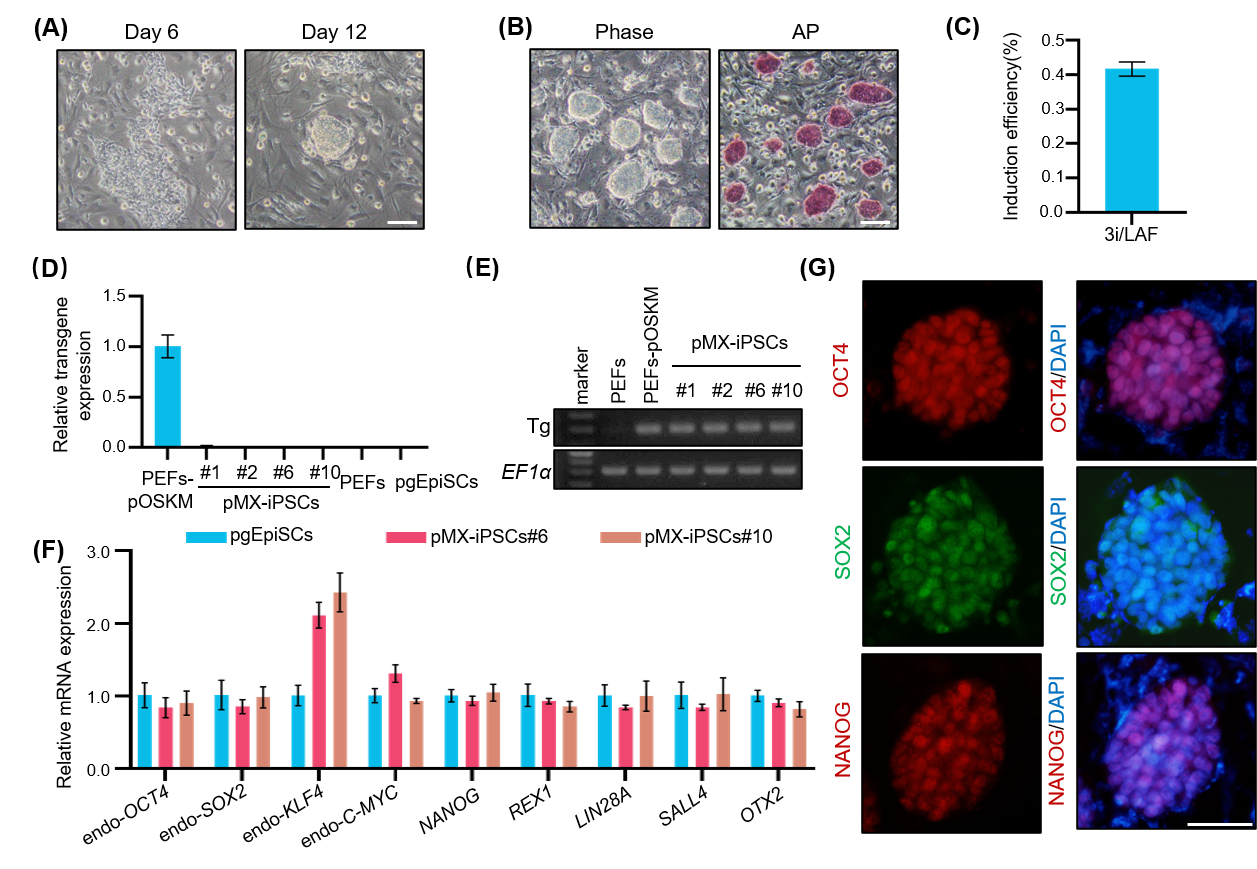


**Figure S2. Generation of retrovirus-silenced iPSCs from PEFs.**

**A,** Reprogramming process of generating pMX-iPSCs. Scale bar, 100 μm.

**B,** Colony morphology and AP staining of pMX-iPSCs. Scale bar, 100 μm.

**C,** Induction efficiency of iPSCs from PEFs using retroviral vector.

**D,** qPCR using primer specifically detecting the retroviral transcript. The PEFs infected with pOSKM virus for three days were used as control.

**E,** Genomic PCR for retroviral vector in pMX-iPSCs.

**F,** qPCR of pluripotency genes in pMX-iPSCs.

**G,** IF staining of key pluripotency markers OCT4, SOX2, and NANOG in pMX-iPSCs#6**.** Scale bar, 50 μm.

For C, D, and F, the error bar indicates ± SD (*n*=3, independent experiments). The experiments in A, B, E, and G were repeated independently three times with similar results.


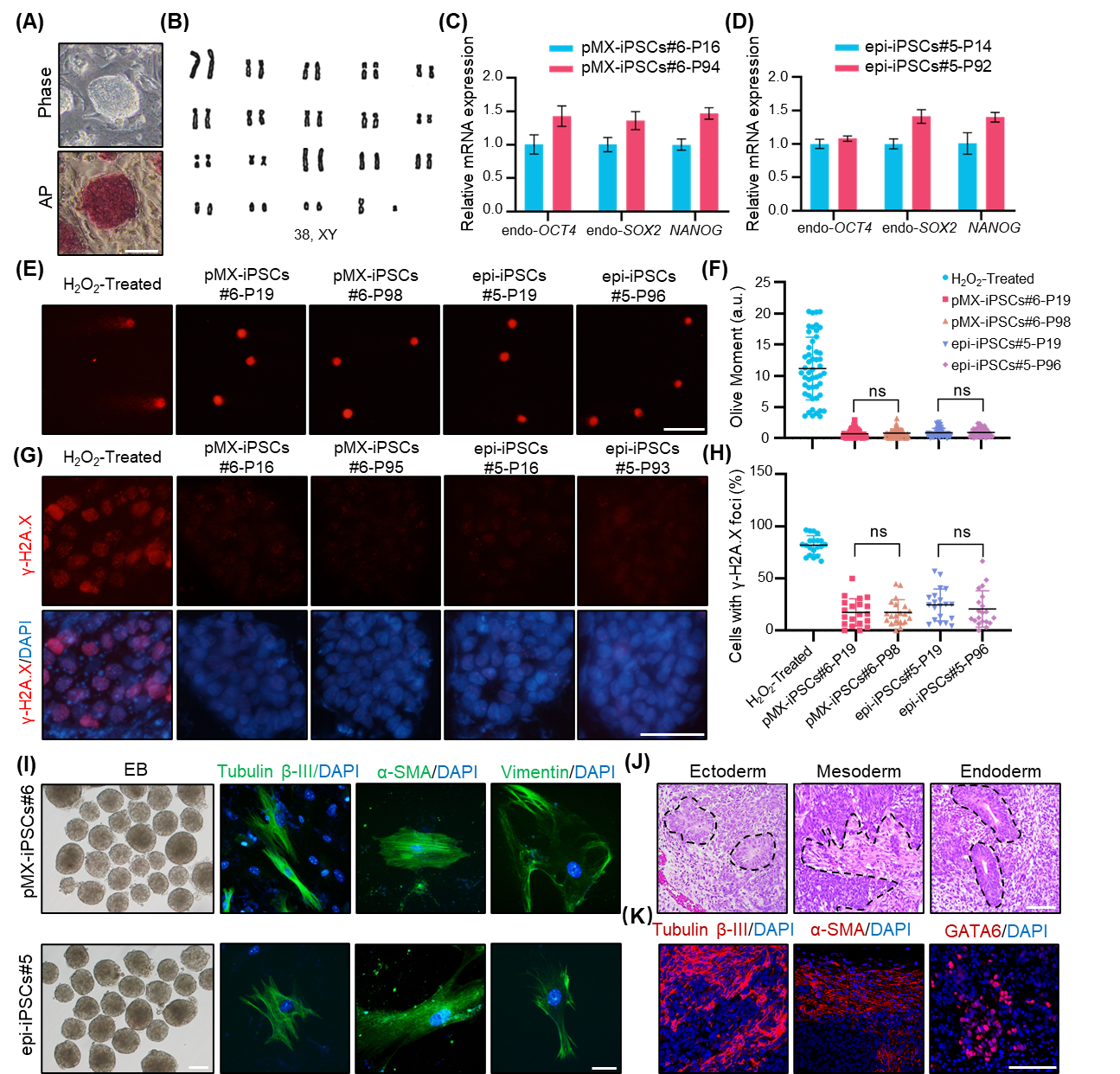


**Figure S3. Pluripotency characteristics of 3i/LAF-iPSCs. Related to Figure 3.**

**A,** Morphology of pMX-iPSCs#6 at passage 95 (P95). Scale bar, 100 μm.

**B,** Karyotype analysis of pMX-iPSCs#6 at passage 97 (38, XY).

**C-D,** Expression of pluripotency genes in pMX-iPSCs#6 (C) and epi-iPSCs#5 (D) at different passages, the error bar indicates ± SD (*n*=3, independent experiments).

**E-F,** DNA damage levels of iPSCs at different passages by the comet assay. Representative images (E) and quantification of the mean olive tail moment were shown (F) (n = 50 nuclei). ns, *P* ≥ 0.05. Scale bar, 100 μm. epi-iPSCs#5-P19 treated with 200 μM H_2_O_2_ were used as positive control.

**G-H,** IF staining of γ-H2A.X for iPSCs at different passages. Representative images (G) and the ratio of cells with γ-H2AX foci are shown (H) (n=20 fields), ns, *P* ≥ 0.05. Scale bar, 50 μm. epi-iPSCs#5-P16 treated with 200 μM H_2_O_2_ were used as positive control.

**I,** *In vitro* EB assays of pMX-iPSCs#6 and epi-iPSCs#5. Scale bar, 100 μm.

**J,** H&E staining of teratomas derived from pMX-iPSCs#6. Scale bar, 100 μm.

**K,** IF staining of teratomas derived from pMX-iPSCs#6. Scale bar, 100 μm.

The experiments in A, B, E, F, G, H, I, J and K, were repeated independently three times with similar results.


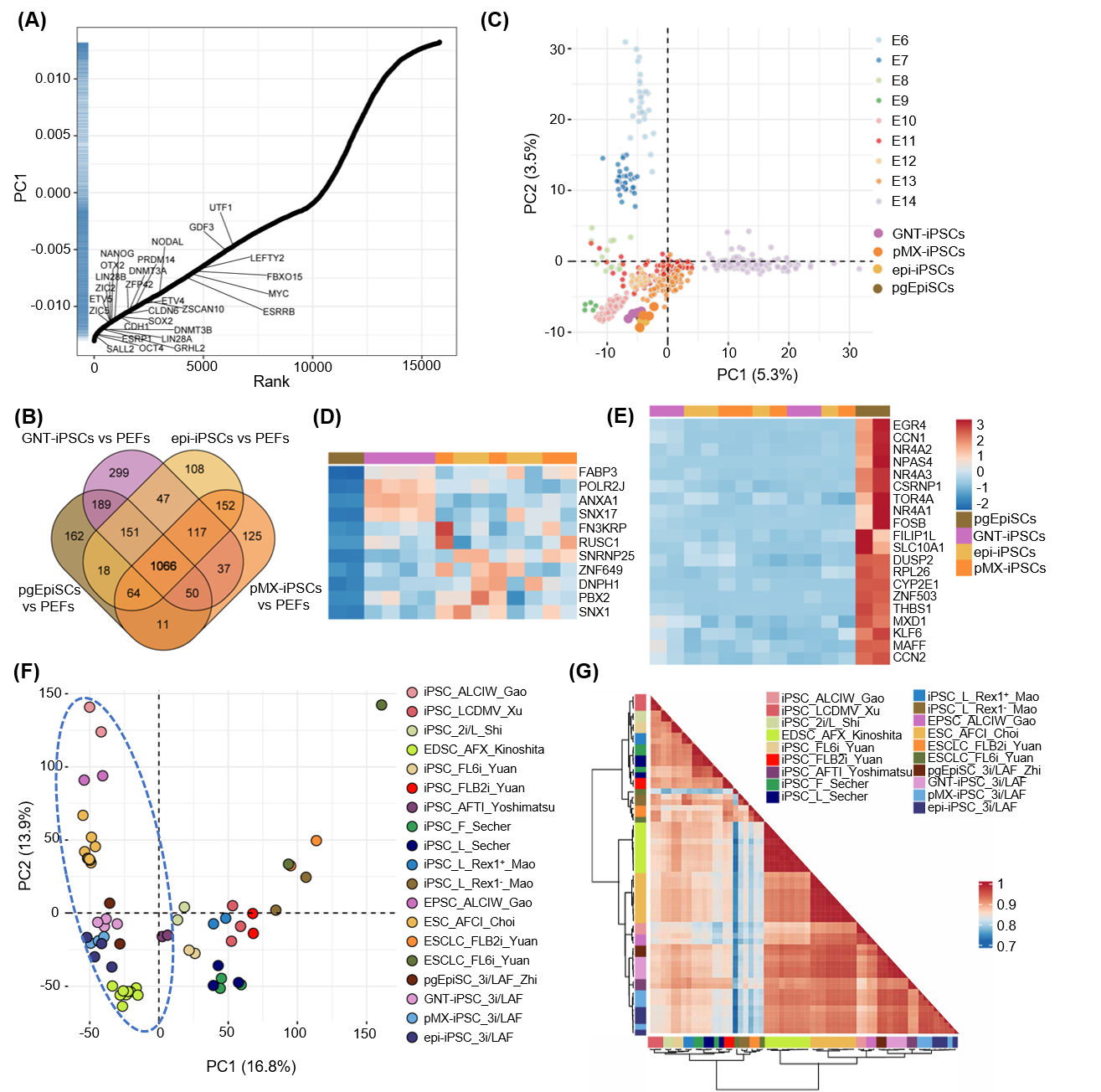


**Figure S4. RNA-seq analysis of 3i/LAF-iPSCs. Related to Figure 4.**

**A,** Loading scores of the pluripotency genes expressed in PC1.

**B,** Venn diagram of upregulated DEGs in 3i/LAF-iPSCs vs PEFs and pgEpiSCs vs PEFs.

**C,** PCA plot of *in vivo* E6-E14 epiblast datasets and 3i/LAF-PSC datasets.

**D-E,** Heatmap of DEGs between 3i/LAF-iPSCs and pgEpiSCs, co-upregulated (D) and co-downregulated (E) genes in 3i/LAF-iPSCs.

**F,** PCA plot of 3i/LAF-iPSCs and published established pig PSCs. The circled area included PSCs with similarity.

**G,** Spearman correlation coefficients of 3i/LAF-iPSCs and public established pig PSCs.

**
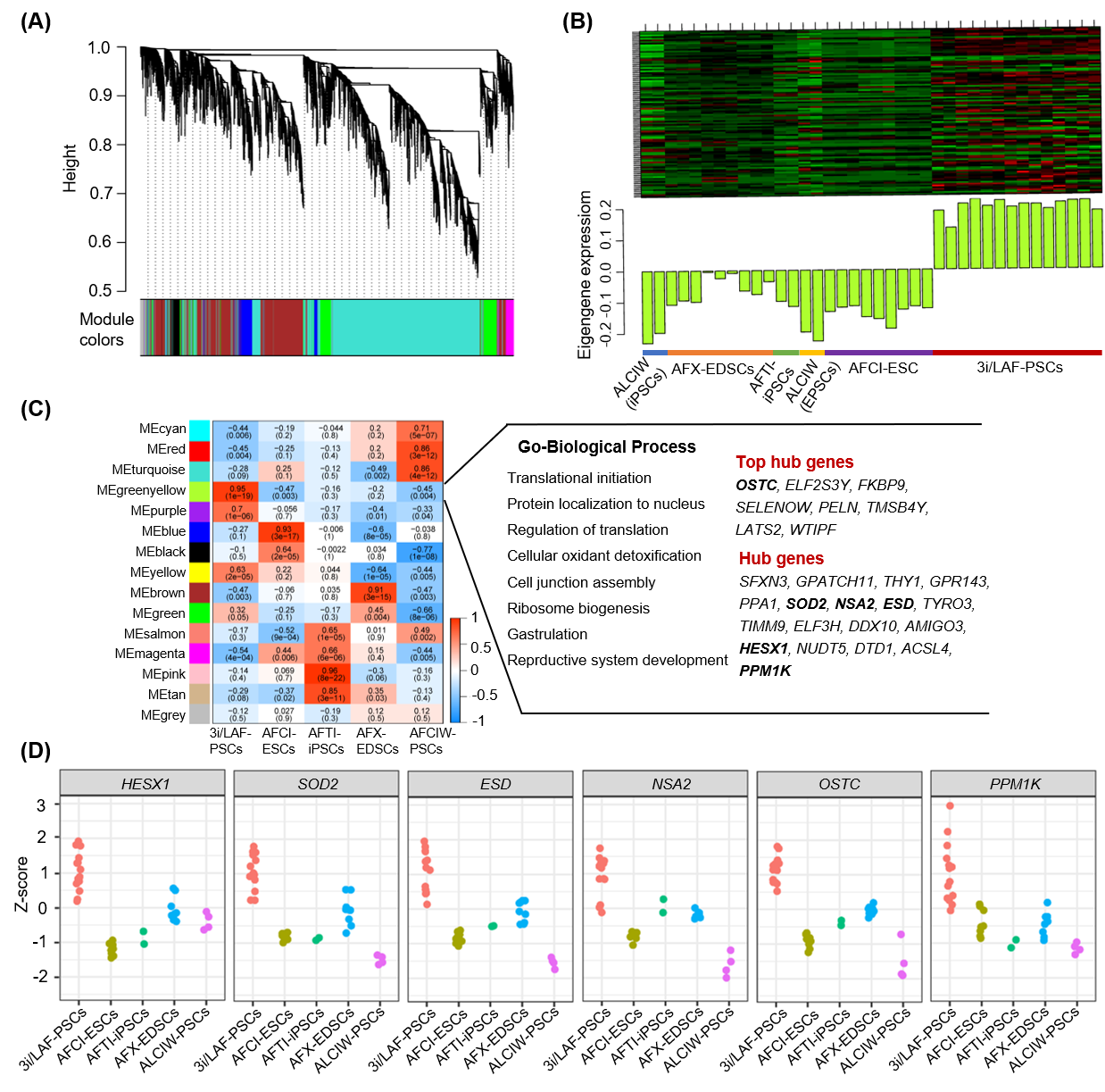
**

**Figure S5. The special transcriptome traits of 3i/LAF-PSCs.**

**A,** Clustering dendrogram of DEGs in different PSCs, with dissimilarity based on topological overlap.

**B,** Clustering heatmap and eigengene expression level of genes in greenyellow module**.**

**C,** Unique profiles of 3i/LAF-PSCs found by WGCNA analysis.

**D,** Expression level of Hub genes for 3i/LAF-PSCs in 3i/LAF-PSCs and other PSCs.

**
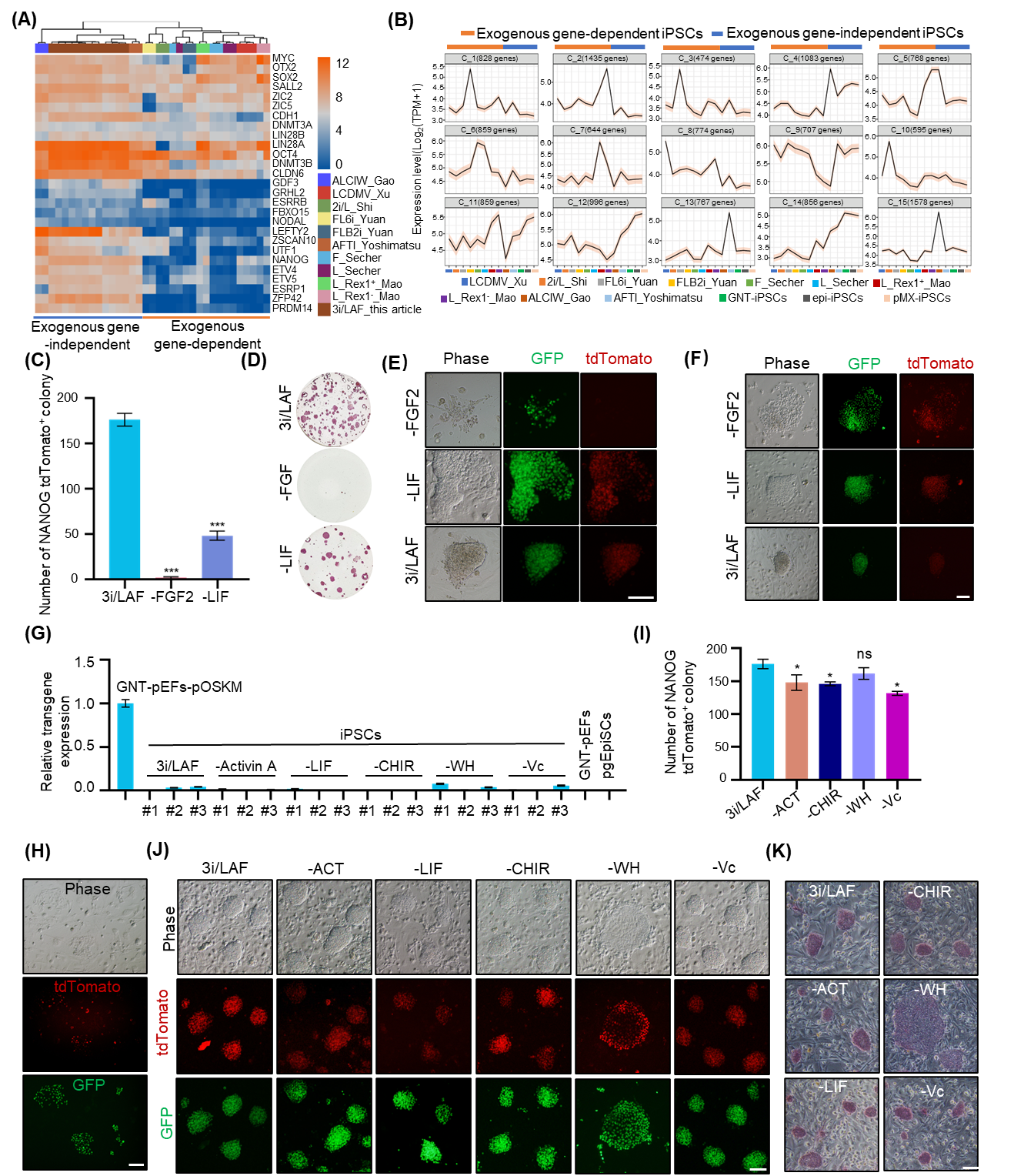
**

**Figure S6. WNT inhibition promotes the establishment of exogenous gene-independent iPSCs. Related to Figure 5.**

**A,** Heatmap of genes related with pluripotency in exogenous gene-independent iPSCs and exogenous gene-dependent iPSCs.

**B,** K-means clustering of the exogenous-gene independent and exogenous-gene dependent iPSC lines.

**C,** Number of NANOG-tdTomato-positive colonies generated from 4×10^4^ GNT-PEFs at day 18 post-infection. Components were removed individually (-FGF2, -LIF), ****P <* 0.001.

**D,** AP staining of reprogrammed original well when reprogrammed under LIF or FGF2 removed media.

**E,** Reprogrammed cells were in a poor proliferative state when cultured in medium without FGF2 at day 12 of reprogramming. Scale bar, 100 μm.

**F,** Colonies generated under medium without FGF2 were loose, Scale bar, 100 μm.

**G,** qPCR using primer specifically detecting the viral transcript in iPSCs under different factor-reduced culture media using primer specifically detecting the viral transcripts.

**H,** Differentiated state of cells picked from medium without IWR-1. Scale bar, 100 μm.

**I,** Number of NANOG-tdTomato-positive colonies generated from 4×10^4^ GNT-PEFs under media without ACT (Activin A), CHIR(CHIR99023), WH(WH-4-023), or Vc (Vitamin C). Components were removed individually.

**J,** Morphology and fluorescence of iPSCs cultured under different media. Scale bar, 100 μm.

**K,** AP staining of iPSCs cultured in media of ACT, LIF, CHIR, WH, or Vc removal. Scale bar, 100 μm.

For C, G, and I, error bar indicates ± SD (*n*=3, independent experiments). The experiments in D, E, F, H, J, and K were repeated independently three times with similar results.


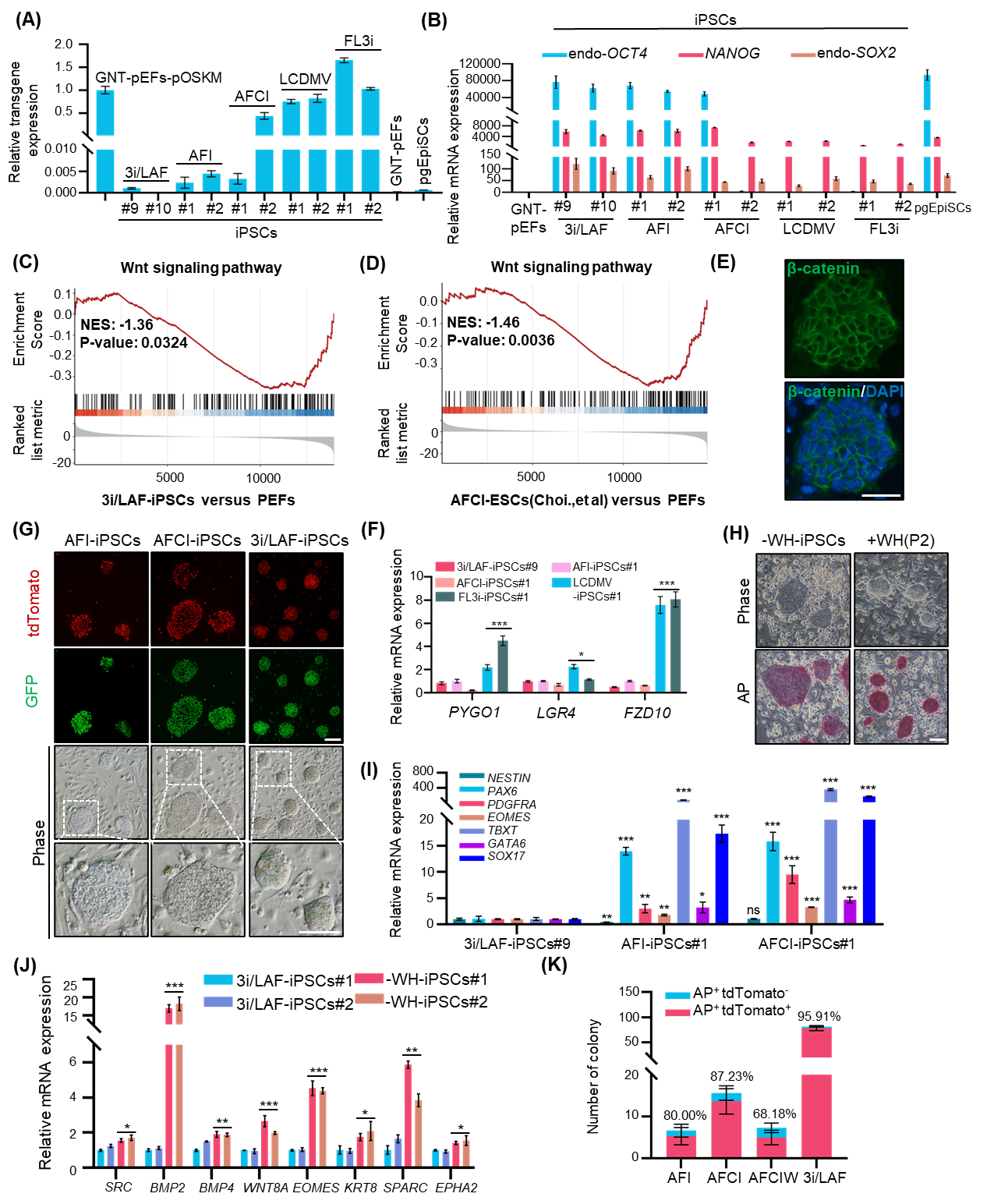


**Figure S7. 3i/LAF system is more stable in terms of reprogramming to establish pluripotency.**

**A,** qPCR using primer specific for retroviral vector in iPSCs reprogrammed under different culture media. The GNT-pEFs infected with pOSKM virus for three days were used as control.

**B,** qPCR of genes related to pluripotency in different iPSC lines.

**C,** GSEA of WNT signaling pathway genes in transcriptome profiles of 3i/LAF-(GNT)-iPSCs and PEFs.

**D,** GSEA of WNT signaling pathway genes in transcriptome profiles of AFCI-ESCs and PEFs.

**E,** IF staining of β-catenin in 3i/LAF-(pMX)-iPSCs. Scale bar, 50 μm.

**F,** qPCR of WNT pathway related genes in exogenous gene-independent iPSCs (3i/LAF-iPSCs, AFI-iPSCs, and AFCI-iPSCs) and exogenous gene-dependent iPSCs (LCDMV-iPSCs and FL3i-iPSCs).

**G,** Morphology of established NANOG-tdTomato-positive iPSC lines under different culture media. Scale bar, 100 μm.

**H,** The flattened morphology returned to normal states after treated with WH-4-023 within two passages (P2). Scale bar, 100 μm.

**I,** qPCR of the lineage markers in iPSC lines generated from different culture media, ns, *P* ≥ 0.05, **P <* 0.05, ***P <* 0.01, and ****P <* 0.001.

**J,** Expression of EMT-related genes by qPCR analysis in iPSCs cultured without WH-4-023. ns, *P* ≥ 0.05, **P <* 0.05, ***P <* 0.01, and ****P <* 0.001.

**K,** Ratio of NANOG-tdTomato-positive colonies generated from different reprogramming systems.

For A, B, F, H, J and K, error bar indicates ± SD (*n*=3, independent experiments). The experiments in E, G, and H were repeated independently three times with similar results.


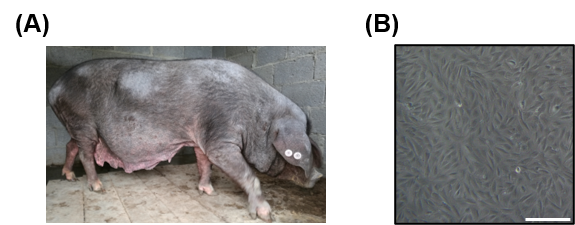


**Figure S8. The state of ear fibroblasts derived from 10-year-old female rare local Wujin fire fair line pig. Related to Figure 6.**

A, Photo of the 10-year-old female rare local Wujin fire fair line pig.

B, Morphology of Wujin-pEFs at passage two. Scale bar, 200 μm.
