# Supplementary material for: Generation of stable integration‐free pig induced pluripotent stem cells under chemically defined culture condition
Source: Cell Prolif. 2023 May 15;56(11):e13487. doi: 10.1111/cpr.13487 (PMC10623960; doi:10.1111/cpr.13487)
Supplement: Supplementary file 2 — Data S1. Supporting information [file CPR-56-e13487-s003.docx]

**Materials and Methods**

**Animal**

CD-1 (ICR) IGS used for isolation of mouse embryonic fibroblasts (MEFs) and BALB/c nude mice for teratoma formation ability test were purchased from Beijing Vital River Laboratory Animal Technology Co., Ltd. (Beijing, China). All mouse experiments were approved by the Institutional Animal Care and Use Committee of China Agricultural University. All pig procedures were approved by the Institutional Animal Care and Use Committee of Yunnan Agricultural University.

**Derivation** **of GNT-pEFs from cloned piglets and culture of the pig fibroblasts**

For derivation of GNT-pEFs, the ear tissues were cut until a homogenate was formed in the Fetal Bovine Serum (FBS)-filled liquid environment. The tissue pieces were smeared evenly to the bottom of the culture plate, then the culture plates were inverted culture for 12 h at 37°C. Wujin-pEFs were separated and provided by Yunnan Agricultural University. The GNT-pEFs were routinely cultured in DMEM medium containing 10% FBS (PEF medium), as well as PEFs and Wujin-pEFs. Fibroblasts were cryopreserved in PEF medium with 40% FBS and 10% DMSO.

**Packaging and collection of virus**

Retrovirus vectors were packaged using pMX-pOKSM^1^ and VSVG. GP2-293 cells cultured in T75 were transfected with 12 μg pMX-pOSKM and 4 μg VSVG using Calcium Phosphate Cell Transfection Kit (Beyotime, C0508). The supernatant was collected after 48 h of transfection and filtered with 0.45 μm filter membrane. 5×PEG8000 was added to the flow-through and left at 4°C for 8 h. The flake precipitation was collected and resuspended in 200 μL Opti-MEM medium.

**Pig iPSCs culture media**

*3i/LAF culture medium (Chemically defined culture medium)*

Pig iPSCs were induced and maintained on MEF feeder layers in 3i/LAF culture medium under 20% O_2_ and 5% CO_2_. The basal medium of 3i/LAF consists of following components per 500 mL: 227.5 mL DMEM/F12 (Thermo Fisher Scientific, 10565-018), 227.5 mL neurobasal (Thermo Fisher Scientific, 21103-049), 2.5 mL N2 supplement (Thermo Fisher Scientific, 17502-048), 5 mL B27 supplement (Thermo Fisher Scientific, 12587-010), 2.5 mL GlutaMAX (Thermo Fisher Scientific, 35050-061), 5 mL nonessential amino acids (Thermo Fisher Scientific, 11140-050), 500 μL β-mercaptoethanol (Thermo Fisher Scientific, 21985-023), 5 mL penicillin-streptomycin (Thermo Fisher Scientific, 15140-122), 25 mL knockout serum replacement (KOSR, Thermo Fisher Scientific, A3181502), and 50 μg/mL ascorbic acid (Vc, Sigma-Aldrich, A4544). Cytokines and small molecules in 3i/LAF contained recombinant FGF-basic (154aa) (10 ng/mL, PeproTech, 100-18-B), recombinant human Activin A (25 ng/mL, PeproTech, 120-14E), recombinant human LIF (10ng/ml, PeproTech, 300-05), CHIR99021 (1 μM, Selleckchem, S1263), IWR-1 (2.5 μM, Selleckchem, S7086), WH-4-023 (1 μM, Selleckchem, S7565). 10 μM Y-27632 (Selleckchem, S1049) was added when passaging, and 2 μM for routinely maintenance.

*Previously described pig culture media*

1) AFCI conditions^2^: 5 ng/mL Activin A, 20 ng/mL FGF2, 1.5 μM CHIR99021, and 2 μM IWR-1;

2) ALCIW conditions^3^: 20 ng/mL Activin A, 5 ng/mL LIF, 5 μM CHIR99021, 5 μM IWR-1, and 0.3 μM WH-4-023;

3) LCDMV conditions^1^: 10 ng/mL LIF, 1 μM CHIR99021, 2 μM (S)-(+)-Dimethindene maleate (DiM, Tocris, 1425), 2 μM Minocycline hydrochloride (MiH, Tocris, sc-203339), and 40 μg/ml Vc.

4) FL3i conditions^4^: 3 μM CHIR99021, 1 μM PD0325901, and 2 μM SB431542 (Selleckchem, S1067).

5) For AFI conditions, 20 ng/mL Activin A, 10 ng/ml FGF2, and 2 μM IWR-1 were used, which was slightly modified from AFTI culture medium.^5^

**Generation of pig iPSCs from GNT-pEFs and PEFs using retroviral vector.**

When GNT-pEFs and PEFs^1^ coverage reached around 70% for 24 h after passage, cells were incubated in PEF medium containing pOSKM virus suspension and 8 μg/mL polybrene. GNT-pEFs and PEFs were replated to 6 well plates with feeder layers three days post-infection at a density of 4×10^4^ cells per well. The PEF medium was changed to 3i/LAF induction medium after 24 h. Domed and smooth-edged colonies were picked on day 16. The iPSCs were digested in StemPro™ Accutase™ Cell Dissociation Reagent (Gibco, A1110501). The cells were then transferred to a 48 well feeder layer-plated plate and grown in 3i/LAF medium. Colonies picked from GNT-EF induction well were NANOG-tdTomato-positive and the colonies picked from PEF induction well were chosen randomly. The culture medium was changed every 24 hours. Cells were cryopreserved in FBS with 10% DMSO.

**Vector construction**

For improving the reprogramming efficiency of episomal vector system, pEV-pig LIN28A and pEV-pig NANOG were constructed based on pEV-hKLF4.^6^ Briefly, pEV-hKLF4 was linearized using *Kpn*Ⅰand *Not*Ⅰ. Pig LIN28A and pig NANOG segments were individually amplified by PCR from pig pgEpiSC cDNA using overlapping PCR primers, then assembled with linearized pEV backbone. The annealed *MSTN* sgRNA sequence was cloned into linearized pGL3-U6-sg-EGFP vector’s *Bsa*Ⅰsite (Addgene, 51133). The primers utilized in this procedure are presented in Table S1 (Key Resources Table) in Supporting information. The vector structures were verified by Sanger sequencing.

**Generation of pig iPSCs from PEFs and Wujin-pEFs using episomal vectors**

Episomal iPSC Reprogramming Kit, including pEV-hOCT4-2A-hSOX2, pEV-hMYC, pEV-hKLF4, and pEV-hBCL2L1 was purchased from Precision BioMedicals Co., Ltd (611005). The induction process was modified based on the manufacturer’s instructions. PEFs were chosen as initiating cells as GNT-pEFs have genomic contamination. Briefly, PEFs were passaged for one time to ensure that cells were in a stable and rapid proliferation state. When the cells expanded to cover 80-85% of the surface area of culture dish, 2 μg pEV-hOCT4-2A-hSOX2, 1 μg pEV-hC-MYC, 1 μg pEV-hKLF4, and 0.5 μg pEV-hBCL2L1 plasmids were transfected into 5×10^5^ cells at 340 V, 1 ms, 3 pulses using a BTX ECM2001 instrument (Harvard Bioscience, Holliston, MA, USA). Transfected cells were replated to 6 well plates with feeder layers, according to 1×10^5^ cells per well. PEF medium was used within 24 h and replaced with PEF and 3i/LAF medium (1:1) the next day. 3i/LAF medium was used after 48 h. For Wujin integration-free iPSCs induction, 1 μg pEV-pig LIN28A and 1 μg pEV-pig NANOG plasmids were added to above kit.

**AP Staining**

Pig iPSCs were washed by DPBS and fixed with 4% paraformaldehyde (Sangon Biotech, 3053589-4) at room temperature for 1 min. After washing 3 times with DPBS, cells were incubated in 500 μL Fast Red Violet (FRV) solution, 250 μL naphthol AS-BI phosphate solution, and 250 μL deionized water for 10 min at 37°C. Then the solution was replaced with DPBS, followed by observation under the microscope.

**Analysis of cell growth, doubling time, and single cell cloning efficiency**

2×10^5^ pig iPSCs were cultivated in 12 well plate with a feeder layer. The cells were digested to single cells and counted by a Luna™ Automated Cell Counter every 12 hours. Each time point contains triplicate cell samples. The cell doubling time was calculated as previous described.^7^ For analysis of single cell cloning efficiency, 200 single cells were picked and cultivated in 24 well plate with feeder layer. About 1 weeks later, the colonies were stained by AP. The number of AP positive colonies were counted for further calculation.

**DNA/RNA extraction, PCR and qPCR**

Cell genome DNA and total RNA were extracted using extraction Kits respectively (TIANGEN, DP304-03 and DP403) following the manufacturer’s instructions. RNA was reversely transcribed using 5× All-In-One RT Master Mix (Abm, G490). PCR was performed using 2× Es Taq MasterMix (CWbio, CW0690S). qPCR was conducted using 2×RealStar Green Power Mixture (GenStar, A311-05) on a LightCycler 480 II Real Time System (Roche). The relative expression was calculated using the comparative CT (2^−ΔΔCT^) method. The ΔCT was calculated using EF1α as internal control. The primers used in qRT-PCR were listed in Table S1 (Key Resources Table) in Supporting information.

**IF Analysis**

Pig iPSCs were washed with DPBS and fixed with 4% paraformaldehyde (Sangon Biotech, 3053589-4) at room temperature for 30 min, followed by washing three times with DPBS. The cells were permeased for 30 min with 0.05% Triton X-100 (Amresco, 0694) and blocked for 1 h with 3% BSA. Primary antibody against OCT4 (RD system, AF-1759), SOX2 (Santa cruz, sc-365823), NANOG (PeproTech, 500-P236), SSEA1 (Abcam, ab16285), SSEA4 (Abcam, ab16287), TRA-1-60 (CST, 4746), TRA-1-81 (CST, 4745), Phospho-Histone H2A.X (Ser139) (CST, 9718), Tubulin β-III (Abcam, ab18207), α-smooth muscle actin (Abcam, ab5964), Vimentin (Abcam, ab92547), SOX1 (Abcam, ab87775), Brachyury (Abcam, ab20680), GATA6 (CST, 5851), β-catenin (Santa Crus, sc-7963) was diluted using Immunol Staining Primary Antibody Dilution Buffer (Beyotime, P0103). The cells were then washed three times with DPBS for 15 min. Respective secondary antibodies conjugated to Alexa Fluor (Invitrogen) was diluted using Immunol Fluorescence Staining Secondary Antibody Dilution Buffer (Beyotime, P0103). After repeating cleaning by DPBS, the cell nuclei were stained by DAPI. The antibodies information used are listed in Table S1 (Key Resource Table) in Supporting information.

**Bisulfite sequencing**

Genomic DNA was processed with an EZ DNA Methylation-Gold kit (Zymo Research, D5005) according to the manufacturer’s instructions. Regions of interest were amplified by PCR using TaKaRa Taq™ Hot Start Version (TakaRa, R007A). The PCR products were purified with a Gel Extraction Kit (OMEGA, D2500), and then cloned into the pMD-19T vector (Takala, 6063). Sequencing results were analysed using the Quantification tool for Methylation Analysis (QUMA) online (<http://quma.cdb.riken.jp/>).

**Karyotype analyses**

The pig iPSCs were incubated for 2.5 h in fresh 3i/LAF medium containing 15% Colcemid Solution (Gibco, 15210-040) when they were in the proliferation phase. Cell pellets were resuspended in 10 ml of 75 mM KCL solution and incubated for 30 min at 37°C, turning over every 5 minutes. Pre-cooled fresh fixative solution (methanol/glacial acetic acid 3:1) was added at a ratio of 1/10, followed by 8 min centrifugation at 1500 rpm to remove the supernatant. Cells pellets were fixed in 10 ml of fixed solution, and incubated on ice for 30 min, followed by incubating on ice under another 10 ml fixed solution for 60 min. Cell pellets were resuspended using fresh fixative solution, then dropped onto glass slides. After the glass slides were stained using the Rapid Giemsa Staining kit (BBI Life Science, E6073141), they were dried in a 37°C oven.

**Alkaline Comet Assay**

Alkaline Comet assay was performed as previously reported.^8,9^ iPSCs treated with 200 μM H_2_O_2_ for 4 h in advance were used as control. Briefly, iPSCs were digested to single cells using accutase when they were in a state of rapid proliferation at 48 h after passage, followed by seperating feeder cells using differential adherent method. The iPSCs were mixed with low-melt agarose and placed in Comet assay slides. The slides were placed at 4°C in the dark for 30 min for solidification, followed by immersion in lysis buffer (100 mM Na_2_EDTA, 2.5 M NaCl, 1% N-lauroylsarcosine, 1% Triton X-100, 10 mM Tris-base, pH 10.0). Then, slides were washed using DPBS for three times, and incubated in cold electrophoresis buffer (1 mM EDTA, 300 mM NaOH, pH > 13) for 30 min. Electrophoresis was carried out at 25 V, 300 mA for 30 min at 4°C in the dark, followed by incubated the slides with Neutralization buffer (0.4 M Tris-HCl, pH 7.4) for 10 min. Comets were stained with 1×StarGreen safe Nucleic Acid Dye (GenStar, E111-01) for 10 min and images were analysed by CASP comet assay software (Andor Technology).

**Embryoid body differentiation**

For *in-vitro* embryoid body differentiation, pig iPSCs were digested to cell suspension, followed by plated on non-adherent plates in basal medium for 48 h at 37°C with gentle shaking (70 rpm). Smooth-edged and large spheroids were picked for adherent differentiation on a gelatin-plated 4 well plate. Fibroblasts medium was changed every two days. After 7-9 days, fiber-like cells were fixed for further dyeing.

**Teratoma formation**

For the teratoma formation, approximately 1×10^7^ pig iPSCs were collected and injected into the posterior neck of BALB/c nude mice. Teratomas could be seen at the injection sites about four weeks later.

**H&E analysis**

Teratomas were harvested from the subcutaneous layer 6 weeks after injection, then washed by DPBS. The teratomas were cut into 5 mm×5 mm tissue pieces. The tissue pieces were fixed with 4% paraformaldehyde at 4°C for 2 days. Tissue blocks were dehydrated using gradient ethanol (70%, 80%, 90%, 95%, and 100% for 1 h each) treatment, then transferred to xylene. The tissue blocks were embedded in paraffin. The thickness of the section was set at 5 μm. The sections were treated with the mentioned ethanol and xylene in reverse order, then stained with haematoxylin (Sigma–Aldrich, MHS16) and eosin (Sigma–Aldrich, HT110116).

**Directional induced differentiation**

The directional induced differentiation was performed as described in previous study.^7^ Briefly, after 48 h culture for iPSCs, 3i/LAF medium was replaced with differentiation medium. For ectoderm induction, the iPSCs were treated with basal medium consisted of 2.5 μM IWR-1, 5 μM SB431542, and 10 ng/ml FGF2 for next 48 h, followed by 4 μM RA, 10 ng/ml FGF2, and 20 ng/ml Noggin for 48 h. For mesoderm induction, the iPSCs were treated with basal medium with 10 ng/ml BMP4, 50 ng/ml Activin A, and 20 ng/ml FGF2 for 48 h, then changed to 3 μM IWR-1, 5 μM CHIR99021, and 20 ng/ml FGF2 in basal medium for another 48 h. For endoderm induction, the iPSCs were treated with basal medium with 10 ng/ml BMP4, 5 μM SB431542, and 10 ng/ml FGF2 for 4 d, then the cells were passaged at a ratio of 1:1 and fixed until the cells reached 100% coverage.

**Generation of GFP-inserted pig iPSCs and *MSTN* KO pig iPSCs.**

To generate GFP-inserted pig iPSCs, 1 μg PBase helper plasmids and 3 μg PB-CMV-EF1A-GFP-NLS plasmids were transfected into 6×10^5^ pig iPSCs at 220 V, 5 ms, 2 pulses using a BTX ECM2001 instrument. The efficiency of GFP transgene was analysed by Flow Cytometry at 72 h after transfection. The NLS-GFP positive pig iPSCs were sorted for further expanded after three times passage. To generate heterozygous *MSTN* KO iPSCs, 3 μg AncBE4max (provided by Xingxu Huang’ lab at ShanghaiTech University) and 3 μg pGL3-U6-*MSTN* sgRNA-EGFP were electroporated into 2.5×10^5^ pig iPSCs. EGFP-positive colonies were picked and expanded. For genotype testing, genomic DNA was extracted using cell lysis buffer (Invitrogen, AM8723) and the lysates were used as PCR template. The PCR products were sequenced by Sangon Biotech (Shanghai) Co., Ltd to clarify the knock out form of the cell lines. The details of sgRNA sequences are provided in Table S1 (Key Resources Table) in Supporting information.

**Generation of iPSCs cloned embryos**

Pig iPSCs were differentiated to fibroblast-like cells in basal medium containing 10 ng/mL BMP4, 5 μM SB431542, and 10 ng/mL FGF2, then used as donor cells. Nuclear transfer was performed as described.^10^ Briefly, oocytes with three or four layers of cumulus cells were selected and cultured to metaphase II, followed by enucleated using a micromanipulator system. Donor cells with high quality were injected into the perivitelline space of enucleated oocytes. The reconstructed embryos were fused in infusion medium using the Electro Cell Fusion Generator (LF201, Nepa Gene, Chiba, Japan) at 200 V/mm for 20 μs, followed by activation in activation medium at 150 V/mm for 100 μs. The embryos were cultured in PZM-3 medium containing 5 μg/mL cytochalasin B for 2 h at 39°C in a humidified atmosphere of 5% CO_2_, 5% O_2_, and 90% N_2_ and then cultured in PZM-3 medium.

**rRNA-depleted RNA-seq**

Around 1×10^6^ GNT-iPSCs#1/3, pMX-iPSCs#6/10 and epi-iPSCs#5/8, respectively, were collected in duplicate. The cells were lysed in Trizol (Invitrogen). RNA-seq library was constructed using VAHTS Universal V6 RNA-seq Library Prep Kit for Illumina® (Vazyme, NR604-02). PCR production was purified using VAHTSTM DNA Clean Beads (Vazyme, N411-03).

**RNA-seq Data Processing and Analysis**

*Raw data quality control, alignment to the genome and analysis of DEGs*

For the all paired-end libraries from public data and our data, trim_garole (v-0.6.6)^11^ software was used for quality control with the parameters: “-q 25 -j 8 --phred33 --length (reads length)/2 -e 0.1 --stringency 4 --paired”. Then, high quality reads from 12 RNA-seq libraries of our data and 49 libraries of public data were mapped to the reference pig genome (Sscrofa 11.1) using HISAT2 (v-2.1.0)^12^ with default parameters. Expression levels of all genes were quantified as reads counts using FeatureCounts (v-2.0.1)^13^ and only genes containing exons were counted, with the reference annotation Sus_scrofa.Sscrofa11.1.105.gtf downloaded from Ensembl (<http://dec2021.archive.ensembl.org/index.html>). The mitochondrial genes were removed. We calculated the TPM in R for all samples and gene was considered a protein coding gene if its TPM value was more than 0.5 in at least half replicates of all biological replicates. DEGs (Log2FoldChange > 2 and padj < 0.05) of PSCs and PEFs were identified using the DEseq2 tool with Wald-test (v-1.30.1).^14^

*Principal component analysis and fractions calculation*

The PCA plot of all samples was performed using the R package FactoMineR (v-2.4)^15^ and visualization was performed using the R package factoextra (v-1.0.7).^16^ To compare the 3i/LAF-PSCs and pig embryo single cell dataset (accession code: CRA003960, <https://ngdc.cncb.ac.cn/gsa/>), 1500 high variable genes (HVGs) calculate by Seurat (v-4.1.0)^17^ across the dataset were used, our data were projected onto this PCA space using the function “predict” in R and plot by “fviz_add” using package factoextra. The loading scores of PC1 were calculate by “prcomp” function. This part of the analysis was based on the CPM values of the high variable genes. Fractions of identity were calculated using DeconRNASeq package^18^ considering HVGs.

*Correlation* *coefficient matrices and heatmaps*

Correlation coefficient matrices of all PSC samples were generated using Spearman’s correlation for all detected genes by “cor” function. For our data, correlation matrices were generated using Pearson’s correlation coefficient for all detected genes. For the pig PSC samples we chosen, only genes with TPM greater than 5 in at least half of the replicate samples were considered for calculation. R packages pheatmap (v-1.0.12)^19^ was used to display correlation matrices and gene expression matrices.

*Ternary plots*

Ternary plots were produced with the R package ggtern (v-3.3.5)^20^ using the median expression for each selected sample, porcine pluripotency genes were highlighted. Density areas were computed using 2D kernel density estimation.

*Construction of Expression Tendencies*

To systematically explore the differences between the pig exogenous gene-independent and exogenous gene-dependent iPSC samples we chose, we constructed the expression tendencies by median TPM value in the selected samples. We first calculated median TPM value in each sample separately. The median TPM value were rescaled and analysed by the k-means clustering method with parameters k=15 and iter.max=100, grouping the high expression genes with similar tendencies between pig exogenous gene-independent and exogenous gene-dependent iPSC samples into separate clusters. The average and standard deviation of median expression levels for each cluster were calculated to evaluate the performance of clustering. The clusters C_4, C_14, and C_8 represent the characteristics of the system separately.

*Core co-expression Network Analysis of 3i/LAF system*

To obtain the core co-expression network in the 3i/LAF system, we constructed a signed weighted co-expression network using R package WGCNA (v-1.70-3).^21^ We first filtered the TPM matrix by selecting the top 10000 median absolute deviation genes, and measured the pair-wise correlations between these genes. Next, co-expression modules were constructed by using ‘blockwiseModules’ based on the correlation matrix with parameter soft-threshold power set to 12. The “greenyellow” module was identified as the characteristic module of the system. Then we calculated intramodular connectivity for all selective genes using “signedKME” function, the genes that belong to “greenyellow” module, correlation coefficient were greater than 0.85 and connectivity were greater than 0.8 were considered top hub genes, the genes that belong to “greenyellow” module, correlation coefficient were greater than 0.8 and connectivity were greater than 0.8 were considered hub genes.

*Functional Enrichment Analysis*

Functional enrichment analysis of selected genes was performed using Metascape (<http://metascape.org>).^22^ The pig genes were mapped to their human orthologs and human (*Homo sapiens*) was the target species for analysis. Enrichment analyses were performed using all genes in the genome as the background set with GO-BP pathway as ontology sources. Terms with a minimum count ≥ 3, adjusted *P* < 0.01, consided to be significant and similar terms, were grouped into clusters.

*Gene Set Enrichment Analysis*

GSEA was calculated by ClusterProfiler (v-3.18.1) use “fgsea” algorithm.^23-24^ The fold change of all genes were ranked in decreasing order and symbols were converted to entrezid by biomaRt (v-2.46.3)^25^.

**Statistical analysis**

The values reported in the graphs are presented as mean ± SD. Student’s *t* test was used to compare different groups. *P*-value less than 0.05 was considered statistically significant and was displayed as **P*＜0.05, ***P*＜0.01, and ****P*＜0.001.

**References**

1. Xu J, Yu L, Guo J, et al. Generation of pig induced pluripotent stem cells using an extended pluripotent stem cell culture system. *Stem Cell Res Ther*. 2019;10(1):193.

2. Choi KH, Lee DK, Kim SW, et al. Chemically defined media can maintain pig pluripotency network in vitro. *Stem Cell Rep.* 2019;13(1):221-234.

3. Gao X, Nowak-Imialek M, Chen X, et al. Establishment of porcine and human expanded potential stem cells. *Nat Cell Biol.* 2019;21(6):687-699.

4. Zhang M, Wang C, Jiang H, et al. Derivation of novel naive-like porcine embryonic stem cells by a reprogramming factor-assisted strategy. *FASEB J*. 2019;33(8):9350-9361.

5. Yoshimatsu S, Nakajima M, Iguchi A, et al. Non-viral induction of transgene-free iPSCs from somatic fibroblasts of multiple mammalian species. *Stem Cell Rep.* 2021;16(4):754-770.

6. Wen W, Zhang JP, Xu J, et al. Enhanced generation of integration-free iPSCs from human adult peripheral blood mononuclear cells with an optimal combination of episomal Vectors. *Stem Cell Rep*. 2016;6(6):873-884.

7. Zhi M, Zhang J, Tang Q, et al. Generation and characterization of stable pig pregastrulation epiblast stem cell lines. *Cell Res*. 2021;32(2):383-400.

8. Swain U, and Subba Rao K. Study of DNA damage via the comet assay and base excision repair activities in rat brain neurons and astrocytes during aging. *Mech Ageing Dev*. 2011;132(8-9):374-381.

9. Tice RR, Agurell E, Anderson D, et al. Single cell gel/comet assay: guidelines for in vitro and in vivo genetic toxicology testing. *Environ Mol Mutagen.* 2000;35(3):206-221.

10. Wei H, Qing Y, Pan W, et al. Comparison of the efficiency of Banna miniature inbred pig somatic cell nuclear transfer among different donor cells. *PloS One*. 2013;8(2):e57728.

11. Martin M. Cutadapt removes adapter sequences from high-throughput sequencing reads. *EMBnet journal*. 2011;17:10-12.

12. Pertea M, Kim D, Pertea GM, et al. Transcript-level expression analysis of RNA-seq experiments with HISAT, StringTie and Ballgown. *Nat Protoc.* 2016;11(9):1650-1667.

13. Liao Y, Smyth GK, and Shi W. featureCounts: an efficient general purpose program for assigning sequence reads to genomic features. *Bioinformatics.* 2014;30(7):923-930.

14. Love MI, Huber W, and Anders S. (2014). Moderated estimation of fold change and dispersion for RNA-seq data with DESeq2. *Genome Biol.* 2014;15(12):550.

15. Lê S, Josse J, and Husson F. FactoMineR: an R package for multivariate analysis. *J Stat Softw*. 2008;25(1):1-18.

16. Kassambara A, and Mundt F. Package ‘factoextra’. Extract and visualize the results of multivariate data analyses. 2017;76.

17. Ha Y, Hao S, Andersen-Nissen E, et al. Integrated analysis of multimodal single-cell data. *Cell.* 2021;184(13):3573-3587 e3529.

18. Gong T, and Szustakowski JD. DeconRNASeq: a statistical framework for deconvolution of heterogeneous tissue samples based on mRNA-Seq data. *Bioinformatics.* 2013;29(8):1083-1085.

19. Kolde R. pheatmap: Pretty Heatmaps. R package version 1.0. 12. CRAN R-project org/package= pheatmap. 2019.

20. Hamilton NE, and Ferry M. ggtern: Ternary diagrams using ggplot2. *J Stat Softw*. 2018; 87:1-17.

21. Langfelder P, and Horvath S. WGCNA: an R package for weighted correlation network analysis. *BMC Bioinform.* 2008; 9:559.

22. Zhou Y, Zhou B, and Pache L, et al. Metascape provides a biologist-oriented resource for the analysis of systems-level datasets. *Nat Commun.* 2019;10(1):1-10.

23. Yu G, Wang L-G, Han Y, et al. clusterProfiler: an R package for comparing biological themes among gene clusters. *OMICS*. 2012;16(5):284-287.

24. Korotkevich G, Sukhov V, Budin N, et al. Fast gene set enrichment analysis. *BioRxiv*. 2021;060012.

25. Durinck S, Spellman PT, Birney, E, et al. Mapping identifiers for the integration of genomic datasets with the R/Bioconductor package biomaRt. *Nat Protoc*. 2009;4(8):1184-1191.
