# Supplementary material for: Generation of stable integration‐free pig induced pluripotent stem cells under chemically defined culture condition
Source: Cell Prolif. 2023 May 15;56(11):e13487. doi: 10.1111/cpr.13487 (PMC10623960; doi:10.1111/cpr.13487)
Supplement: Supplementary file 3 — Table S1. Key Resources Table [file CPR-56-e13487-s001.docx]

Supplementary information, Table S1, Key Resources Table

| **Antibody used in this study** | | | | | | |
| --- | --- | --- | --- | --- | --- | --- |
| Goat polyclonal anti-OCT3/4 | | RD system | | AF-1759 | | 1:1000 |
| Mouse monoclonal anti-Sox-2 (E-4) | | Santa Cruz | | sc-365823 | | 1:500 |
| Rabbit polyclonal anti-human Nanog | | PeproTech | | 500-P236 | | 1:500 |
| Mouse monoclonal anti-SSEA1(MC480) | | Abcam | | Ab16285 | | 1:500 |
| Mouse monoclonal anti-SSEA4(MC813) | | Abcam | | Ab16287 | | 1:500 |
| Mouse monoclonal anti-human-TRA-1-60 | | CST | | 4746 | | 1:500 |
| Mouse monoclonal anti-human-TRA-1-81 | | CST | | 4745 | | 1:500 |
| Rabbit monoclonal anti-human-Phospho-Histone H2A.X | | CST | | 9718 | | 1:1000 |
| Rabbit polyclonal anti-neuron specific beta III Tubulin | | Abcam | | ab18207 | | 1:250 |
| Rabbit polyclonal anti-alpha smooth muscle Actin | | Abcam | | ab5694 | | 1:250 |
| Rabbit monoclonal anti-Vimentin | | Abcam | | Ab92547 | | 1:250 |
| Rabbit monoclonal anti-GATA-6 (D61E4) | | CST | | 5851 | | 1:1000 |
| Rabbit polyclonal anti-SOX1 | | Abcam | | ab87775 | | 1:1000 |
| Rabbit polyclonal anti-Brachyury/Bry | | Abcam | | ab20680 | | 1:50 |
| Mouse monoclonal Anti-β-catenin | | Santa Cruz | | sc-7963 | | 1:1000 |
| **Chemicals, Peptides, and Recombinant Proteins** | | | | | | |
| Recombinant Human FGF-basic (154 a.a.) | | PeproTech | | | Cat# 100-18B | |
| Human/Murine/Rat Activin A (E.Coli) | | PeproTech | | | Cat# 120-14E | |
| Recombinant Human LIF | | PeproTech | | | Cat# 300-05 | |
| Recombinant Murine BMP-4 | | PeproTech | | | Cat# 315-27 | |
| Recombinant Human Noggin | | PeproTech | | | Cat# 120-10C | |
| All-Trans Retinoic Acid | | PeproTech | | | Cat# 3027949 | |
| Bovine Serum Albumin | | Sigma-Aldrich | | | Cat# A1470 | |
| Ascorbic Acid | | Sigma-Aldrich | | | Cat# A4544 | |
| CHIR99021 | | Selleckchem | | | Cat# S1263 | |
| IWR-1 | | Selleckchem | | | Cat# S7086 | |
| WH-4-023 | | Selleckchem | | | Cat# S7565 | |
| Y-27632 | | Selleckchem | | | Cat# S1049 | |
| SB431542 | | Selleckchem | | | Cat# S1067 | |
| Neurobasal™ Medium | | Thermo Fisher | | | Cat# 21103-049 | |
| DMEM/F-12, GlutaMAX™ supplement | | Thermo Fisher | | | Cat# 10565-018 | |
| Neurobasal™ Medium | | Thermo Fisher | | | Cat# 21103-049 | |
| DMEM/F-12, GlutaMAX™ supplement | | Thermo Fisher | | | Cat# 10565-018 | |
| KnockOut™ DMEM | | Thermo Fisher | | | Cat# 10829-018 | |
| N-2 Supplement (100×) | | Thermo Fisher | | | Cat# 17502-048 | |
| B-27™ Supplement (50×),  minus vitamin A | | Thermo Fisher | | | Cat# 12587-010 | |
| GlutaMAX™ Supplement | | Thermo Fisher | | | Cat# 35050-061 | |
| MEM NEAA Solution (100×) | | Thermo Fisher | | | Cat# 11140-050 | |
| 2-Mercaptoethanol | | Thermo Fisher | | | Cat# 21985-023 | |
| Bovine Serum Albumin（BSA） | | Sigma | | | Cat# 9048-46-8 | |
| Penicillin-Streptomycin  (10,000 U/mL) | | Thermo Fisher | | | Cat# 15140-122 | |
| Gelatin (0.1% in water) | | Stem Cell Technologies | | | Cat# 07903 | |
| Trypsin-EDTA (0.05%), phenol red | | Gibco | | | Cat# 25300120 | |
| DMEM, high glucose, L-glutamine, Sodium Pyruvate | | Gibco | | | Cat# 8122494 | |
| Fetal Bovine Serum | | VISTICH | | | Cat# SE100-B | |
| Accutase cell dissociation reagent | | Gibco | | | Cat# A11105-01 | |
| TrypLE™ Express | | Gibco | | | Cat# 12605010 | |
| Dulbecco's phosphate-buffered saline (DPBS) | | Gibco | | | Cat# C14190500 | |
| Trizol | | Invitrogen | | | 15596026 | |
| **Critical Commercial Kits** | | | | | | |
| RNAprep pure Cell / Bacteria Kit | | TIANGEN | | | Cat# DP430 | |
| Hifair^®^ III 1st Strand cDNA Synthesis SuperMix for qPCR (gDNA digest plus) | | YEASON | | | Cat# 11141ES60 | |
| TIANamp Genomic DNA Kit | | TIANGEN | | | Cat# DP304 | |
| Rapid Giemsa staining kit | | BBI Life Sciences | | | Cat# E202FA0001 | |
| Gel & PCR Clean Up Kit | | OMEGA | | | Cat# D2000-02 | |
| Endo-free Plasmid Mini Kit | | OMEGA | | | Cat# D6950-02B | |
| 2× RealStar Green Power Mixture | | GenStar | | | Cat# A311-05 | |
| Episomal iPSC Reprogramming Kit | | Precision BioMedicals Co.,Ltd | | | Cat# 611005 | |
| Immunol Staining Blocking Buffer | | P0102 | | | Beyotime | |
| Immunol Fluorescence Staining Primary Antibody Dilution Buffer | | P0103 | | | Beyotime | |
| Immunol Fluorescence Staining Secondary Antibody Dilution Buffer | | P0108 | | | Beyotime | |
| Calcium Phosphate Cell Transfection Kit | | C0508 | | | Beyotime | |
| EZ DNA Methylation-Gold kit | | Zymo Research | | | D5005 | |
| VAHTS Universal V6 RNA-seq Library Prep Kit for Illumina® | | Vazyme | | | NR604-02 | |
| **qRT-PCR primer sequences used in this study** | | | | | | |
| Name | Forward | | Reverse | | | |
| Retroviral transgene  (ex-OCT4) | GACGGCATCGCAGCAGCTTGGATACAC | | GAGAAGGCGAAGTCGGAAG | | | |
| endo-*OCT4* | CTTCACCACCCTGTACTCCTC | | GCTTCTCTCCCTAGCTCACC | | | |
| total-*OCT4* | GGCGATCCCAGGACATCAAA | | AGCTGCAAAGCCTCAAAACG | | | |
| endo-*SOX2* | CATCAACGGTACACTGCCTCTC | | ACTCTCCTCCCATTTCCCTCTTT | | | |
| total-*SOX2* | CCAGAAGAACAGCCCAGACC | | GCTTCTCCGTCTCCGACAAA | | | |
| endo-*KLF4* | AGGCACTACCGCAAACATACT | | CCAGGGAGACAGTGTGAAAGG | | | |
| total-*KLF4* | AAAAGGACGGCCACTCACAC | | GCCCAGTATGTTTGCGGTAG | | | |
| endo-*C-MYC* | AGGAGCAAAAGCTCGTGTCA | | CCAGCCAAGGTTGTGAGGTT | | | |
| total-*C-MYC* | AAAAGGTCGGAATCGGGGTC | | GTTTCTCCTCTGGCGTTCCA | | | |
| *NANOG* | CCTACAATCCAGCTCTTTGG | | CTCAGGCATTGGTGAAGATT | | | |
| *REX1* | TCTGAACCCCTCGTGGAAGA | | AGCTTGCTGTAAGCACCTGT | | | |
| *LIN28A* | GTTCTGCATTGGGAGCGAGA | | GGCAGTTTGCATTCCTTGGC | | | |
| *SALL4* | ATCGACGTTTATCCGAGCCC | | TGAGAAGTTCTTCCCGCACC | | | |
| *OTX2* | CACTGTTTGCTAAGACCCGATACC | | GACCTCCATTCTGCTGTTGTTGC | | | |
| *WNT8A* | CTGCAGAACAGCCACAACAC | | CACCACATGCCTACACTGGT | | | |
| *WNT5A* | CGCGAAGACAGGCATCAAAG | | CCTATCTGCATGACCCTGCC | | | |
| *LPR5* | CAGCGTGGAGGAGTTCTCAG | | GGAGGCTCACCACAAGTCAA | | | |
| *SRC* | GCCAACATCCTGGTTGGAGA | | ATCCCAAAGGACCACACGTC | | | |
| *BMP4* | TTCATTTTAGGAGCCATTCTGTAGT | | TCCTAGCAGGACTTGGCATAAT | | | |
| *EOMES* | ACTCCCATGGACCTCCAGAA | | TCGCTTACAAGCACTGGTGT | | | |
| *TBXT* | GAAGTACGTGAACGGGGAGT | | CACGATGTGGATTCGAGGCT | | | |
| *NESTIN* | GGGACCTTTGCTCTCTGTCC | | AAGGCTGGCATAGGTGTGTC | | | |
| *PAX6* | TGTCCAACGGATGTGTGAGT | | TCTGTCTCGGATTTCCCAA | | | |
| *PDGFRA* | GGTCACCTGTGCCGTCTTTA | | TTTGATGGACGGGACCTTGG | | | |
| *SOX17* | CTGCACTTTGTGTGCAAGCC | | TAATACACTGCGGAGCTGGC | | | |
| *BMP2* | GTCTTTCGGGAGCAGACACA | | GTCACCAACCTGGTGTCCAA | | | |
| *KRT8* | TCGCACGAAGACGGAGATTT | | CTGAGCATCCTTGACAGCCA | | | |
| *SPARC* | ATCGGCGAGTTTGAGAAGGT | | TGCAAGGTCCGATGTAGTCC | | | |
| *EPHA2* | CCCGACGAGATCACGGTTAG | | CAGCTCGGGACACTTCTTGT | | | |
| *PYGO1* | TGCCACAGATACATGCACCA | | GGTATCACAGCCCCACACTG | | | |
| *LGR4* | GCAGTGGGTTCTTGCCAAAG | | GCCTGCACCTGATCAGAACT | | | |
| *FZD10* | TGACCGGTGTCTGTTACGTG | | TACTCCACGTTGAGGCGTTC | | | |
| *EF1α* | AATGCGGTGGGATCGACAAA | | CACGCTCACGTTCAGCCTTT | | | |
| **PCR primer sequences used in this study** | | | | | | |
| Name | Forward | | Reverse | | | |
| NANOG-5’arm  knock in-test | TGTCCATTGCTGAAGCATGTAAT | | GAAGTTAGTAGCTCCGCTTCCTG | | | |
| NANOG-3’arm  knock in-test | GCAACCTCCCCTTCTACGAG | | GTGAAGCACACGGTAGGGTA | | | |
| pEV-pLIN28A | GACTGAGTCGCCCGGGTACCATGGGCTCTGTGTCAAACCAG | | TCTGCTCGAGCGGCCGCATTCTGAGCCTCTGGGAGCA | | | |
| pEV-pNANOG | GACTGAGTCGCCCGGGTACCATGAGTGTGGATCCAGCTTGT | | TCTGCTCGAGCGGCCGCCATATCTTCAGGCTGTATGTTCATGGAGTAAT | | | |
| EBNA1 | TTTAATACGATTGAGGGCGTCT | | GGTTTTGAAGGATGCGATTAAG | | | |
| WPRE | GGTTTAAACGCGTCGACAAT | | GTTGCGTCAGCAAACACAGT | | | |
| V-*hOCT4* | ATGTGGTCCGAGTGTGGTTC | | CAGAGTGGTGACGGAGACAG | | | |
| V-*hSOX2* | AGCAGACTTCACATGTCCCA | | TCATTTGCTGCCAGATCCTC | | | |
| V-*hKLF4* | GAAATTCGCCCGCTCAGATG | | TCATTTGCTGCCAGATCCTC | | | |
| V-*hC-MYC* | TCCTCCGACAGACTGAGTC | | CCTCCTCGTCGCAGTAGAAA | | | |
| V-*hBCL2L1* | TCCTCCGACAGACTGAGTC | | CTCCATCTCCGATTCAGTCCC | | | |
| V-*pNANOG* | TCCTCCGACAGACTGAGTC | | GTCGAGGGTCTCAGCAGAT | | | |
| V-*pLIN28A* | GAGCATCAACCATATGGTGG | | TCATTTGCTGCCAGATCCTC | | | |
| pig OCT4 demy | TATGGGGGATTTGTATTAAGGG | | CCAATAAAACCAAAACTCTCCA | | | |
| pig NANOG demy-outer | AATAAGTTTGAATTGGAGATTTAAAG | | ATCTCCTCCAAATATTAAAAATATCA | | | |
| pig NANOG demy-inner | TGAGGTTGGTAGATAGGATTAATTG | | AAAATAAAATAAAATAAATCACCCTT | | | |
| **PCR primer sequences used in *MSTN* Gene Editing** | | | | | | |
| *MSTN*-sgRNA | ACCGTCTGCCAAATACCAGTGCCT | | AAACAGGCACTGGTATTTGGCAGA | | | |
| *MSTN*-sg-test | GTAAAGGCCCAACTGTGGAT | | TCAGTAAACTCTTACCAGCCCA | | | |
| **Deposited Data** | | | | | | |
| GNT-iPSCs RNA-seq data | | | This article | | | |
| pMX-iPSCs RNA-seq data | | | This article | | | |
| epi-iPSCs RNA-seq data | | | This article | | | |
| PEFs | | | Our lab^1^ | | | |
| pgEpiSCs | | | CRA003960 | | | |
| pEPSCs_Gao RNA-seq data | | | E-MTAB-7253^2^ | | | |
| piPSCs_Gao RNA-seq data | | | E-MTAB-7253^2^ | | | |
| pESCs_Choi RNA-seq data | | | GSE120031^3^ | | | |
| pESCLCs_Yuan RNA-seq data | | | GSE126150^4^ | | | |
| piPSCs_Yuan RNA-seq data | | | GSE126150^4^ | | | |
| piPSCs_Shi RNA-seq data | | | GSE143484^5^ | | | |
| piPSCs_Xu RNA-seq data | | | GSE141935^1^ | | | |
| piPSCs_Secher RNA-seq data | | | GSE92889^6^ | | | |
| piPSCs_Mao RNA-seq data | | | GSE87361^7^ | | | |
| EDSCs_Kinoshita | | | GSE172420^8^ | | | |
| piPSCs_ Yoshimatsu | | | GSE152493^9^ | | | |

**References**

1. Xu J, Yu L, Guo J, et al. Generation of pig induced pluripotent stem cells using an extended pluripotent stem cell culture system. *Stem Cell Res Ther*. 2019;10(1):193.

2. Gao X, Nowak-Imialek M, Chen X, et al. Establishment of porcine and human expanded potential stem cells. *Nat Cell Biol.* 2019;21(6):687-699.

3. Choi KH, Lee DK, Kim SW, et al. Chemically defined media can maintain pig pluripotency network in vitro. *Stem Cell Rep.* 2019;13(1):221-234.

4. Yuan, Y., Park, J., Tian, Y., et al. A six-inhibitor culture medium for improving naive-type pluripotency of porcine pluripotent stem cells. *Cell Death Discov.* 2019;5(1):104.

5. Shi B, Gao D, Zhong L, et al. IRF-1 expressed in the inner cell mass of the porcine early blastocyst enhances the pluripotency of induced pluripotent stem cells. *Stem Cell Res Ther.* 2020;11(1):505.

6. Secher JO, Ceylan A, Mazzoni G, et al. Systematic in vitro and in vivo characterization of Leukemia-inhibiting factor- and Fibroblast growth factor-derived porcine induced pluripotent stem cells. *Mol Reprod Dev.* 2017;84(3):229-245.

7. Mao J, Zhang Q, Deng W, et al. Epigenetic modifiers facilitate induction and pluripotency of porcine iPSCs. *Stem Cell Rep*. 2017;8(1):11-20.

8. Kinoshita M, Kobayashi T, Planells B, et al. Pluripotent stem cells related to embryonic disc exhibit common self-renewal requirements in diverse livestock species. *Development*. 2021;148(23).

9. Yoshimatsu S, Nakajima M, Iguchi A, et al. Non-viral induction of transgene-free iPSCs from somatic fibroblasts of multiple mammalian species. *Stem Cell Rep.* 2021;16(4):754-770.
